# Acceptability of and Willingness to Take Digital Pills by Patients, the Public, and Health Care Professionals: Qualitative Content Analysis of a Large Online Survey

**DOI:** 10.2196/25597

**Published:** 2022-02-18

**Authors:** Astrid Chevance, Axel Fortel, Adeline Jouannin, Faustine Denis, Marie-France Mamzer, Philippe Ravaud, Stephanie Sidorkiewicz

**Affiliations:** 1 Center for Research in Epidemiology and Statistics Université de Paris-French National Institute for Health and Medical Research Paris France; 2 Service d'épidémiologie clinique Hôpital Hôtel Dieu Assistance Publique- Hôpitaux de Paris Paris France; 3 Faculté de médecine de Créteil Université Paris-Est-Créteil Créteil France; 4 Département de Médecine Générale Université de Rennes Rennes France; 5 Centre d’investigation clinique de Rennes Centre Hospitalo-Universitaire de Rennes Université de Rennes-French National Institute for Health and Medical Research Rennes France; 6 Department of Ethics, Research, Translations Centre de Recherche des Cordeliers, Sorbonne Université, Université de Paris French National Institute for Health and Medical Research Paris France; 7 Department of Psychiatry Groupe Hospitalo-Universitaire Paris Psychiatrie Neurosciences Paris France; 8 Medical ethics Unit Hôpital Necker Enfants-Malades Assistance Publique-Hôpitaux de Paris Paris France; 9 Department of Epidemiology Columbia University Mailman School of Public Health New York, NY United States; 10 Département de Médecine Générale Université de Paris-French National Institute for Health and Medical Research Paris France

**Keywords:** acceptability, health technology assessment, clinical effectiveness research, ethics, digital pill, digital health, digital therapeutics, ingestible sensor, adherence

## Abstract

**Background:**

Digital pills are pills combined with a sensor, which sends a signal to a patch connected to a smartphone when the pills are ingested. Health care professionals can access patient data from digital pills online via their own interface, thus allowing them to check whether a patient took the drug. Digital pills were developed for the stated goal of improving treatment adherence. The US Food and Drug Administration approved the first digital pills in November 2017, but the manufacturer withdrew its application to the European Medicines Agency in July 2020 because of insufficient evaluation.

**Objective:**

As recommended for the evaluation of health technologies, this study assesses the prospective acceptability of and willingness to take digital pills among patients, the public, and health care professionals.

**Methods:**

Participants were patients who were receiving long-term treatment for a chronic condition, public participants (both groups recruited from a representative sample), and health care professionals. Participants answered 5 open-ended questions regarding the acceptability of digital pills and 1 close-ended question regarding the willingness to take digital pills, which were developed in a preliminary qualitative study. We explored the 5 theoretical dimensions of acceptability by performing an abductive qualitative content analysis of all free-text responses. We assessed data saturation with mathematical models. We fitted a multivariate logistic regression model to identify the sociodemographic and health characteristics associated with the willingness to take digital pills.

**Results:**

Between January 29, 2020, and April 18, 2020, 767 patients, 1238 public participants, and 246 health care professionals provided 11,451 free-text responses. We identified 98 codes related to the acceptability of digital pills: 29 codes on perceived clinical effectiveness (eg, sensor safety cited by 66/2251 participants, 29.5%), 6 on perceived burden (eg, increased doctors’ workload, 164/2251 participants, 7.3%), 25 on perceived ethicality (eg, policing, 345/2251 participants, 15.3%), 30 codes on perceived opportunity (eg, exclusively negative perception, 690/2251 participants, 30.7%), and 8 on affective attitude (eg, anger, 541/2251, 24%). Overall, 271/767 (35.3%) patients, 376/1238 (30.4%) public participants, and 39/246 (15.8%) health care professionals reported willingness to take digital pills. This willingness was associated with male sex (odds ratio 1.98, 95% CI 1.62-2.43) and current use of a connected device to record health settings (with a dose–response relationship).

**Conclusions:**

The prospective acceptability of and willingness to take digital pills were limited by clinical and ethical concerns both at the individual and societal level. Our results suggest that digital pills should not be considered a mere change in the form of drug administration but a complex intervention requiring specific evaluation before extended use in clinical routine practice as well as an ethical and legal framework to ensure safe and ethical collection and use of health data through a patient-centered approach.

## Introduction

More than one-third of people worldwide live with at least 2 chronic conditions; hence, an increasing number of people are receiving long-term treatment [1]. Control of many of these conditions depends on adherence to treatment, which can be defined as the degree to which the use of medication by the patient corresponds with the prescribed regimen [2]. However, about half of patients with chronic conditions do not take their medications as prescribed. Poor treatment adherence leads to a range of negative consequences such as poor control of disease, life-threatening events, treatment resistance, and increasing costs [3-8]. In 2003, the World Health Organization suggested that improving treatment adherence would have a greater impact than any improvement in specific medical treatments, and this has led to a continuous search for effective interventions to monitor and improve medication adherence (eg, blood drug dosage, self-reported patient diaries, smart pill dispensers) [2,9,10].

The most recent monitoring tool, approved in 2017 by the US Food and Drug Administration, is called a “digital pill.” Digital pills are pills combined with a sensor, which sends a signal to a patch connected to the smartphone of the patient when the pill is ingested. Health care professionals and caregivers can access patient data (eg, adherence, activity, heart rate) from digital pills online via their own interface, thus allowing them to check whether a patient took the drug. The sensor is an ingestible event marker made from a copper–magnesium pair of electrodes within a silicon insulating skirt disk 5 mm in diameter and 0.3 mm thick. On contact with gastric fluid, the sensor sends a unique digital code to allow identification and timestamping of the medication and dose form [11]. The first digital pill was an antipsychotic (aripiprazole) used for schizophrenia or bipolar disorder treatment, which was rapidly followed by a range of other digital pills for multiple medical conditions (eg, antihypertension, diabetes, viral diseases) [12].

Digital pills have been developed for the stated goal of improving medication adherence and optimizing treatment management; these are used to identify nonadherence to first-line therapies before more expensive second-line therapies can be considered [10,13]. The European Medicines Agency declared that the evaluation for the digital pill submitted was inadequate and withdrew the application on July 26, 2020; the quality of the available studies (observational studies or small trials) and the lack of clinical relevance of the outcomes (short-term outcomes related to adherence to digital pills, safety outcomes restricted to tolerance of the patch) were points of criticism that led to this withdrawal [12,14-17].

Moreover, guidelines for the evaluation of health technologies recommend taking into account patient and public perspectives, which has not been done yet for digital pills [18]. Acceptability is a multifaceted construct that reflects the extent to which people delivering or receiving a health care intervention consider it appropriate based on anticipated, present, or retrospective responses to the intervention [19]. It includes the extent to which the intervention is perceived likely to achieve its purpose (perceived clinical effectiveness), the amount of effort required to participate in the intervention (perceived burden), the extent to which the intervention aligns with individual values (perceived ethicality), the extent to which benefits/values must be compromised to engage in the intervention (perceived opportunity), and how people feel about the intervention (affective attitude) [19].

As part of new health technology assessment, this study aims at evaluating the prospective acceptability of and willingness to take digital pills by 3 subpopulations (patients, public participants, and health care professionals).

## Methods

We conducted an online survey by recruiting a representative sample of the French general population, dichotomized into patients and public participants, and an additional convenience sample of health care professionals. The survey included 5 open-ended questions related to the acceptability of digital pills and 1 close-ended question regarding the willingness to take digital pills (Multimedia Appendices 1 and 2).

### Participants and Recruitment

Patients were adults (≥18 years old) with at least one self-reported chronic condition for >6 months and who were prescribed long-term treatment for >1 month. Public participants included adults (≥18 years old) who did not meet the eligibility criteria to be classified as patients. Health care professionals were professionals prescribing, delivering, or monitoring pharmaceutical treatments (physicians, nurses, pharmacists, and midwives). All participants were living in France, spoke French, and had access to the internet.

We used the 2 distinct methods to recruit participants:

- The representative sample was recruited using a quota sampling method in the IPSOS Access Panel [20]. Quotas were based on the known profile of the French population in terms of sex, age, socioprofessional category, and population density of the residential area. This sample was secondarily classified into patients and public participants according to the eligibility criteria. We calculated weights with the rim weighting method—raking (Multimedia Appendices 3) [21].
- The additional convenience sample of health care professionals was recruited via an online campaign by sending emails to professional associations and posting advertisements on social networks (Multimedia Appendices 4).

All participants provided informed electronic consent before enrollment. The Institutional Review Board CERAPHP.5, Paris, France, gave ethical approval (IRB0001198).

To recruit patients with various combinations of chronic conditions and socioeconomic status, perform quantitative analysis using logistic regression, and ensure that data saturation was reached based on previous experience with an online survey and considering the fact that 34.5% of the French population lives with a chronic condition, we targeted a representative sample of 2000 participants [22,23]. Regarding the convenience sample of health care professionals, we decided to stop recruitment after reaching 95% data saturation [22].

### Survey Development and Data Collection

We conducted a preliminary qualitative study to draft the questions (wording, formatting, and content) of the survey and provide the investigators with a contextual framework for analyzing and interpreting textual data. A researcher trained in qualitative methods (AC) conducted face-to-face collaborative interviews with 5 patients, 5 public participants, and 3 health care professionals (Multimedia Appendix 5). From the results of this preliminary study, we developed an illustrated explanation of digital pills, 5 open-ended questions on the acceptability (Textbox 1) and 1 close-ended question on the willingness to take digital pills (“Would you agree to use this device for yourself?”) (Multimedia Appendices 1 and 2).

**Open-ended questions to all participants on the acceptability of digital pills.**

- What do you think of this device (transmitter + Bluetooth patch + smartphone app + access to data)? We want to know your immediate reaction: try to write down all the ideas that came to you when you first saw this device.
- What do you envisage are the positive aspects of this device (transmitter + Bluetooth patch + smartphone app + access to data)? For example, in what situation(s)/for whom could it be useful, what kind of benefit could it bring?
- What do you envisage are the negative aspects of this device (transmitter + Bluetooth patch + smartphone app + access to data)? For example, what drawbacks or risks do you foresee?
- If your doctor suggested a treatment using this device (transmitter + Bluetooth patch + smartphone app + access to data), what would your reaction be? What would you think of his/her approach?
- The manufacturer says that this device will allow for greater consistency between the prescription and the actual taking of medications. In your opinion, why would a person take their treatment more consistently if they were equipped with this device?

In addition, we collected sociodemographic data (eg, sex, age, level of education) for patients and public participants, professional status data (profession and duration of professional experience) for health care professionals, and health characteristics for all participants (eg, chronic condition, current use of a connected device to collect health data) (Multimedia Appendices 1 and 2).

The survey for patients and public participants was available online in a dedicated IPSOS platform. The survey for health care professionals was available in a LimeSurvey form. Both surveys were tested online for usability, clarity, and wording in a convenience sample of 10 patients, 20 public participants, and 5 health care professionals, whose feedback helped improve the final version.

### Analyses

#### Acceptability of Digital Pills

We conducted a multiple-round qualitative content analysis of each free-text response by following a 3-step approach: inductive open coding of all units of meaning related to the acceptability of digital pills, inductive condensation of the codes, and inductive development of the themes and deductive classification of the themes in the 5 dimensions of acceptability [19,24].

In the first step, the team of coders (AC, AF, AJ, FD) read and reread the responses at the individual level (all responses to all questions individual by individual) and at the question level (all responses for all individuals question by question). The process of coding identified in each response units of meaning and assigned a code. A unit of meaning was defined as any expression in the text related to the acceptability of digital pills, as explained by Sekhon et al [19]. The coding was limited to the manifest content of the responses, except for the first open-ended question on the emotional reaction to the digital pill, for which the latent emotional content was interpreted when possible [25]. During a face-to-face meeting, all researchers coded together all responses for 100/2005 (5%) individuals of the representative sample and 50/246 (20%) individuals in the sample of health care professionals. Then, 1 researcher coded all responses (AC) and 3 others double-checked the codes (AF, AJ, FD). In case of discrepancies, consensus was reached by discussion with another researcher (SS). To reduce interpretation bias, coders had different backgrounds and training (psychiatry, family medicine, training in clinical research, social science, cognitive psychology, and medical ethics).

The second and third steps occurred in a meeting in which the 4 coders and SS inductively condensed all the codes using semantic, psychological, and anthropological considerations. Then, they categorized the codes inductively into themes and classified them deductively into the 5 theoretical dimensions of acceptability [19].

We then calculated the number of citations for each code identified with the qualitative content analysis in each group using the weighted data for patients and public participants.

Data saturation was assessed for patients, public participants, and health care professionals with a mathematical model [22].

#### Willingness to Take Digital Pills

We calculated the number and proportion of participants who agreed to take digital pills. We estimated odds ratios (ORs) and 95% CIs using the weighted data of the representative sample to calculate the association of the willingness to take digital pills with all socioeconomic and health characteristics. We used the same data for a multivariate logistic regression model, including sex, age, socioprofessional status, residential area, chronic condition, current use of a connected device to collect health data, and ease to talk about treatment with the doctor. For the regression, we considered the response “I do not wish to answer” to be a refusal to take digital pills. The significance threshold for *P* values was calculated with Bonferroni correction to correct for multiple testing.

All quantitative analyses involved using the free software R v3.3 (R Foundation for Statistical Computing), and the package “survey.”

### Data Sharing Statement

The deidentified quantitative data set will be shared on request (astrid.chevance@gmail.com).The textual data set will not be shared because of ethical restrictions.

## Results

**Figure 1:**
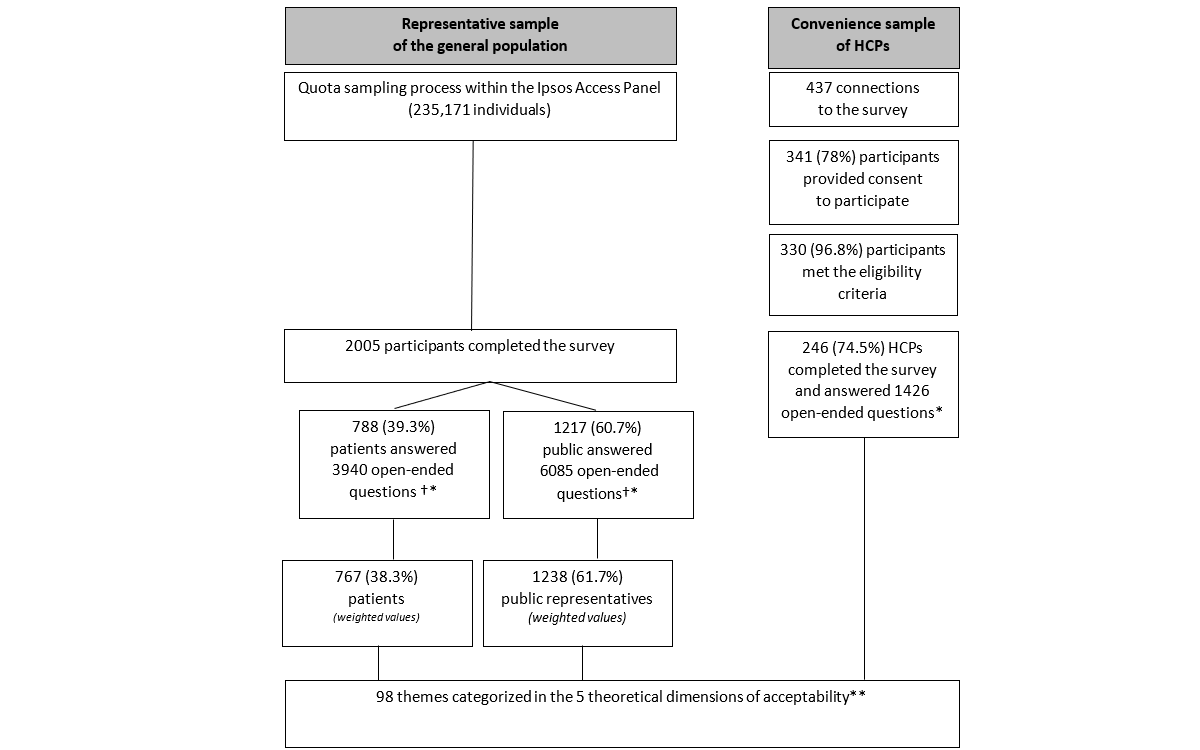
Flowchart. HCP: health care professional. †Unweighted numbers and proportions of patients and public representatives. *Participants answered 5 open-ended questions (panel 1). We added the number of open-ended questions answered for each type of participant. **Results presented in Figure 3 and Multimedia Appendix 10.

Between January 29, 2020, and February 5, 2020, IPSOS recruited a representative sample of 2005 participants (767 patients, 38.3%; 1238 public participants, 61.7%) (Figure 1). We recruited a convenience sample of 246 health care professionals between February 4, 2020, and April 18, 2020.

### Participants

Among patients, 396/767 (51.6%) were women and the mean age was 54.6 (SD 15.6) years (Table 1). Hypertension was the most reported chronic condition (169/767, 22%), followed by diabetes (135/767, 17.6%) and chronic pain (81/767, 10.6%) (Multimedia Appendix 6).

**Table 1:** Sociodemographic and health characteristics of the representative sample and subgroups of patients and public participants.

Sociodemographic and health characteristics		Total (N=2005), n (%)		Patients^a^ (n=767), n (%)		Public participants (n=1238), n (%)
**Sex**						
	Female	1050 (52.4)	396 (51.6)		654 (52.8)	
	Male	955 (47.6)	371 (48.4)		584 (47.2)	
**Age (years)**						
	18-34	519 (25.9)	116 (15.1)		403 (32.6)	
	35-49	470 (23.4)	128 (16.7)		342 (27.6)	
	50-64	601 (30)	285 (37.2)		316 (25.5)	
	>65	415 (20.7)	238 (31)		177 (14.3)	
**Highest level of education**						
	Secondary school or under	75 (4.7)	43 (5.6)		52 (4.2)	
	Youth training	386 (19.3)	175(22.9)		210 (16.9)	
	High school graduate	471 (23.5)	172 (22.3)		299 (24.1)	
	Two-year university degree	458 (22.8)	174 (22.7)		284 (22.9)	
	Bachelor’s degree (BA, BS)	341 (17)	122 (15.9)		219 (17.7)	
	Master’s degree or beyond	254 (12.7)	80 (10.4)		174 (14.1)	
**Socioprofessional category**						
	Farmers	18 (0.9)	6 (0.8)		12 (1)	
	Self-employed professional workers	74 (3.7)	28 (3.6)		46 (3.7)	
	Senior managers	198 (9.9)	58 (7.6)		141 (11.4)	
	Technicians and associate professionals	305 (15.2)	68 (8.9)		236 (19.1)	
	Employees	349 (17.4)	89 (11.6)		260 (21)	
	Manual workers	265 (13.2)	76 (9.9)		188 (15.2)	
	Retired people	559 (27.9)	322 (42)		237 (19.1)	
	Unemployed	163 (8.1)	103 (13.4)		61 (4.9)	
	Student	74 (3.7)	17 (2.2)		57 (4.6)	
**Population density of the residential area (inhabitants)**						
	Rural city (<2000)	451 (22.5)	171 (22.3)		280 (22.6)	
	2000-19,999	360 (18)	138 (18)		211 (17)	
	20,000-99,999	276 (13.8)	109 (14.2)		161 (13)	
	≥100,000	605 (30.2)	229 (29.9)		377 (30.5)	
	Paris agglomeration	329 (16.4)	120 (15.6)		209 (16.9)	
**Number of visits to a doctor in the past year**						
	>10 times	208 (10.4)	140 (18.3)		68 (5.5)	
	5-10 times	590 (29.4)	336 (43.8)		254 (20.5)	
	<5 times	1046 (52.2)	286 (37.3)		759 (61.3)	
	Have not seen a doctor this year	152 (7.6)	5 (0.6)		148 (12)	
	Do not wish to answer	9 (0.4)	0 (0)		9 (0.7)	
**Ease to talk about the treatment with the doctor (eg, adherence, adverse events)**						
	Easy	1767 (88.1)	696 (90.8)		1071 (86.5)	
	Difficult	214 (10.7)	70 (9.1)		144 (11.6)	
	Do not wish to answer	24 (1.2)	1 (0.1)		23 (1.9)	
**Skipped the long-term treatment during the past month**						
	Never	N/A^b^	496 (64.7)		N/A	
	Once a week	N/A	198 (25.8)		N/A	
	Several times a week	N/A	54 (7)		N/A	
	Almost every day	N/A	15 (1.9)		N/A	
	Never started the prescribed treatment	N/A	3 (0.4)		N/A	
	Do not wish to answer	N/A	2 (0.2)		N/A	
**Checking health settings recorded by connected devices**						
	Daily	220 (11)	99 (12.9)		120 (9.7)	
	Weekly	213 (10.6)	85 (11.1)		128 (10.3)	
	Monthly	143 (7.2)	59 (7.7)		84 (6.8)	
	Rarely	257 (12.8)	99 (12.9)		159 (12.8)	
	Never	1168 (58.2)	425 (55.4)		743 (60)	
	Do not wish to answer	4 (0.2)	0 (0)		4 (0.3)	
**Agree to take a digital pill**						
	Yes	647 (32.3)	271 (35.3)		376 (30.4)	
	No	1261 (62.9)	461 (60.1)		800 (64.6)	
	Do not wish to answer	97 (4.8)	35 (4.6)		62 (5)	

^a^Chronic condition was defined as at least 1 long-term treatment and 1 self-reported condition. The most common chronic conditions were hypertension, diabetes, chronic pain, thyroid disease, heart disease, asthma, and dyslipidemia. All chronic conditions are presented in Multimedia Appendix 6.

^b^N/A: not applicable.

A total of 99/767 (12.9%) patients and 120/1238 (9.7%) public participants reported checking health data recorded by a connected device daily, and 524/767 (68.3%) patients and 902/1238 (72.9%) public participants reported rarely checking or never checking data (Table 1).

Among health care professionals, there were 86/246 (35%) general practitioners, 50/246 (20.3%) psychiatrists, and 40/246 (16.3%) nurses (Table 2; Multimedia Appendix 7).

**Table 2:** Sociodemographic and health characteristics of the health care professionals (N=246).

Sociodemographic characteristics		Health care professionals, n (%)
Age (years), mean (SD)		35.5 (10.3)
**Sex**		
	Female	155 (63)
	Male	90 (36.6)
	Other	1 (0.4)
**Occupation**		
	Medical doctor	183 (74.4)
	Nurse	40 (16.3)
	Pharmacist	8 (3.2)
	Midwife	9 (3.7)
	Other	6 (2.4)
**Medical specialists^a^**		
	General practitioner	86 (47)
	Psychiatrist	50 (27.3)
	Cardiologist	7 (3.8)
	Other medical specialists^b^	40 (21.2)
Professional experience (years), mean (SD)		10.5 (10)
**Has a chronic condition^c^**		
	Yes	28 (15.4)
	No	218 (88.6)
**Checking health settings recorded by the connected devices**		
	Every day	11 (4.5)
	Several times per week	25 (10.2)
	Several times per month	16 (6.5)
	Rarely	60 (24.4)
	Never	133 (54)
	Do not wish to answer	1 (0.4)
**Assessment of patient medication adherence**		
	Rarely	21 (8.5)
	Depending on the patient and the medical situation	113 (46)
	Systematically for each patient with long-term treatment and at each consultation	112 (45.5)
**Agree to take a digital pill**		
	Yes	39 (15.8)
	No	166 (67.5)
	Do not wish to answer	11 (4.5)
	Missing data	30 (12.2)

^a^n=183.

^b^For clarity, we report the results for only the 3 most prevalent medical specialties. The other medical specialists were anesthetists, intensive care unit physicians, emergency physicians, child psychiatrists, surgeons, obstetricians*-*gynecologists, hematologists, neurologist, gastroenterologists, dermatologists, endocrinologists, oncologists, pneumologists, infectious diseases specialists, rheumatologists, nephrologists, pediatric care specialists, palliative care specialists.

^c^Chronic condition was defined as at least 1 long-term treatment and 1 self-reported condition.

### Acceptability of Digital Pills

**Figure 2:**
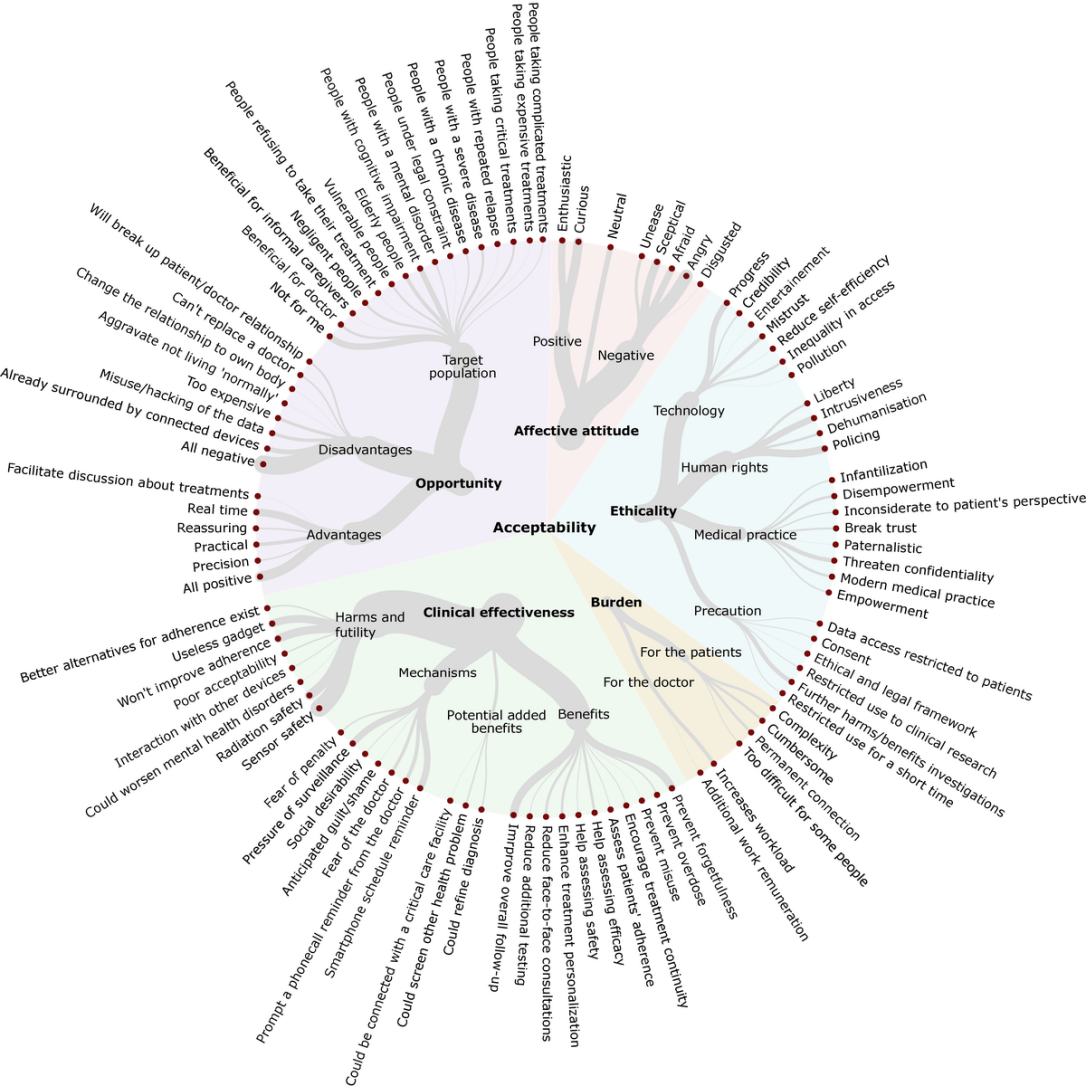
Acceptability of connected drugs. Dots represent codes regarding acceptability of treatments identified by the qualitative content analysis of the responses for 767 patients, 1238 public participants, and 246 health care professionals. The width of the line is proportional to the number of spontaneous citations of the codes by the representative sample of patients and public participants (N=2005). The overarching categories in bold correspond to the 5 dimensions of the theoretical model of acceptability by Sekhon et al.

The qualitative content analysis of the 11,451 open-text responses identified 98 codes related to acceptability. Data saturation was reached for patients (99.4%), public participants (100%), and health care professionals (97.7%) (Multimedia Appendix 8). We classified the 98 codes into the 5 theoretical dimensions of acceptability: perceived clinical effectiveness (29 codes, 29%), perceived burden (6 codes, 6%), perceived ethicality (25 codes, 25%), perceived opportunity (30 codes, 30%), and affective attitude (8 codes, 8%) [19]. We report the most-cited codes for each dimension with the proportion of citation and participants’ quotes in Table 3. Figure 2 and Multimedia Appendix 9 report all 98 codes.

**Table 3:** Quotes of patients, public participants, and health care professionals that illustrate some of the codes regarding acceptability of digital pills.

Code	Patients (n=767)	Public participants (n=1238)	HCPs^a^ (n=246)	Examples of quotes
Congruent with value of progress	59 (7.7)	131 (10.6)	29 (11.8)	“I will try it because I have the culture of new technologies.” (Public participant, man, 34 years old) “I am for, because I believe in modernity and artificial intelligence.” (Public participant, man, 43 years old) “Only by accepting certain methods can one advance in medicine.” (Nurse, woman, 46 years old)
Congruent with value of credibility	12 (1.6)	30 (2.4)	9 (3.7)	“It's interesting, it's as close to reality as possible and probably the truth.” (Public participant, man, 31 years old) “There will no longer be any doubt about taking or forgetting to take medication, and the monitoring by the doctor that imposes honesty on the patient.” (Public participant, man, 39 years old) “This helps to avoid the medical errors that often occur when a patient lies about taking their medication.” (Public participant, man, 29 years old)
Intrusiveness (conflict of value with privacy)	66 (8.6)	150 (12.1)	62 (25.2)	“This is spying from inside my body. I would be worried about the side effects too.” (Patient, woman, 64 years old) “There is far too much indiscretion and invasion of privacy.” (Public participant, woman, 55 years old) “It’s horrible! It feels as if it’s really touching on intimacy. Ethically, it is frightening. That we use applications is one thing because we can limit its use in, in time, but a pill that we ingest is really to futuristic and too invasive.” (Public participant, woman, 33 years old) “It is a categorical refusal, I still prefer to take a drug that has less effect but I will never consciously swallow a capsule that allows other people to follow me or my lifestyle, even if already in society we are monitored in many areas without knowing it, my doctor has the right to suggest it after it is up to the person to accept it or not” (Patient, woman, 44 years old)
Dehumanization of patients	32 (4.2)	46 (3.7)	41 (16.7)	“We're already guinea pigs and we're becoming robots... “(Patient, man, 64 years old) “When you're sick, you're already dispossessed of your 'medical' life, especially in a hospital environment, so with that on top of it, no thanks.” (Patient, woman, 53 years old) “No thanks, I won't take any connected medication, I don't want to be kept under surveillance that much. It contributes to the dehumanisation of our society.” (Neurologist, woman, 29 years old)
Inconsiderate to patient’s perspective	25 (3.3)	21 (1.7)	30 (12.2)	“Very bad idea, we're not in a dictatorship. People are free to take care of themselves or not. Even the sick are free.” (Patient, man, 75 years old) “Policing, (no) more freedom for the patient to stop a treatment that seems to be harming him without being observed.” (Public participant, woman, 40 years old) “A medication such as you're presenting it to me makes me think of forced treatment. If we refuse to take the medication or forget about it will our doctor contact us to explain ourselves?” (Patient, woman, 25 years old)
Improves overall follow-up	105 (13.7)	170 (13.7)	9 (3.7)	“It allows us to monitor the effectiveness, which is already good! And also, that the doctor cares a little bit about his patient through closer monitoring.” (Patient, man, 57 years old) “The family doctor or specialist may monitor the illness on a day-to-day basis, whether good or bad, and may modify treatment or dosage if there are any problems, undesirable side effects or worsening of the patient's health. In addition, this device could also alert to new complications or illnesses that were not previously detected and that could be managed more quickly.” (Public participant, man, 74 years old)
Poor acceptability	24 (3.1)	22 (1.8)	42 (17)	“That's the problem: if a person doesn't follow a prescription, why would they want it to be known that they're doing whatever.” (Patient, woman, 59 years old) “In psychiatry, this process can be complicated for patients who are very often suspicious and persecuted, and all the more so if ‘something enters their body’ to keep them under surveillance... For the elderly, connected tools are not news except for the next generation.” (Nurse, woman, 51 years old) “I don't really see the point of such a complex system, when most of the information can be gathered through interrogation. The patients who are going to accept this device will probably be compliant patients and not the most problematic ones. Compliance is also a matter of education and not ‘policing’. I don't see how being constantly kept under surveillance is going to get the patient to take their medication, other than by telling them they're going to get shouted at by their doctor, which is not our role.” (Dermatologist, woman, 26 years old)
Useful for people with cognitive disorder	87 (11.3)	87 (7)	29 (11.8)	“Unless I'm losing my mind, I wouldn't want to be kept under surveillance all the time.” (Patient, woman, 77 years old) “It might perhaps be useful for animals, but for humans, I don't see it. Unless, the person is not autonomous (e.g., senile dementia).” (Public participant, woman, 31 years old) “For the elderly or people with amnesia: allows for better remote monitoring (and less cost for nurses to travel to the home).” (Patient, woman, 62 years old) “I don't see the point in it for me, but for people who are out of their minds, why not? It's not very moral, but why not put it on for people without asking their opinion...“ (Public participant, woman, 36 years old)

^a^HCPs: health care professionals.

In Figure 2, which shows the acceptability of connected drugs, the dots represent codes regarding acceptability of treatments identified by the qualitative content analysis of the responses for 767 patients, 1238 public participants, and 246 health care professionals. The width of the line is proportional to the number of spontaneous citations of the codes by the representative sample of patients and public participants (N=2005). The overarching categories in bold correspond to the 5 dimensions of the theoretical model of acceptability by Sekhon et al [19]. *Perceived clinical effectiveness* is the extent to which the intervention is likely to achieve its purpose. *Perceived burden* is the amount of effort required to participate in the intervention. *Perceived ethicality* is the extent to which the intervention fits well with individual values. *Perceived opportunity* is the extent to which benefits/values must be given up to engage in the intervention. *Affective attitude* indicates how people feel about the intervention.

### Perceived Clinical Effectiveness

Participants reported perceived benefits (11/98 codes, 11%) and harms (8/98 codes, 8%) and proposed further developments to the device to improve benefits (3/98 codes, 3%). Finally, they determined the underlying mechanisms of digital pills to improve medication adherence (7/98 codes, 7%).

Patients (105/767, 13.7%) and public participants (170/1238, 13.7%) believed that digital pills could improve follow-up. Potential harms cited were adverse events due to sensor ingestion (190/767 patients, 24.8%; 415/1238 public participants, 33.5%) or radiation from wireless technology (102/767 patients, 13.3%; 212/1238 public participants, 17.1%). The underlying mechanisms cited to lead to a potential improvement in adherence included behavioral mechanisms such as a smartphone schedule reminder (96/767 patients, 12.5%; 122/1238 public participants, 9.9%) and the pressure of surveillance (77/767 patients, 10%; 141/1238 public participants, 11.4%).

Out of the 246 health care professionals, 64 (26%) considered digital pills to be useless gadgets, and 60 (24.4%) doubted the safety of the sensor. The mechanisms most cited to lead to improved adherence were the pressure of surveillance (73/246, 29.7%) and fear of arguing with the doctor (44/246, 17.9%).

Participants described the pressure of surveillance as a disciplinary mechanism based on social desirability, fear of being reprimanded, fear of social disqualification as a “bad patient,” and fear of shame or guilt. Therefore, participants raised concerns about a policing patient–doctor relationship and the meaning of care in the future.

The fear of being kept under surveillance; it is being treated like a child, I find it, and even degrading.

Patient, woman, 71 years old

The person would take their treatment because they would know that the doctor has access to the actual taking. It is a social pressure: the doctor has particular expectations that the patient will want to respect.

Patient, woman, 19 years old

### Perceived Burden

Participants distinguished doctors’ burden (2/98 codes, 2%) from patients’ burden (4/98 codes, 4%).

Patients and public participants mostly cited the complexity of the device (32/767, 4.2% and 46/ 1238, 3.7%, respectively) and the wearing of a cumbersome device (34/767, 4.4% and 41/1238, 3.3%, respectively) to be burdensome. Participants (62/767 patients, 8.1%; 65/1238 public participants, 5.3%) also reported the increased workload due to the processing of a large amount of data to be a problem.

Participants (12/767 patients, 1.6%; 14/1238 public participants, 1.1%; 15/246 health care professionals, 6.1%) identified contradictions in digital pills and found it unconvincing. Participants reported that taking medication seemed easier than applying a patch, activating the Bluetooth function, and ingesting the medication, especially for people who needed assistance with taking medication (eg, older people, individuals with cognitive impairment).

There will be better monitoring if the sick person is a mobile phone enthusiast: true for the young, less obvious for the elderly.

Patient, man, 71 years old

I find it hard to imagine someone who needs monitoring of their medication intake (dependent person, elderly, etc...) having such a patch device / smartphone.

Patient, man, 36 years old

### Perceived Ethicality

Participants described whether taking a digital pill aligned with their values related to technology (7/98 codes, 7%), human rights (4/98 codes, 4%), and medical practice (8/98 codes). Participants also suggested precautions to limit the unethical use of digital pills (6/98 codes).

Regarding ethicality of the technology, patients and public participants mentioned that digital pills align with their values related to progress (59/767, 7.7% and 131/ 1238, 10.6%, respectively) and contradicted their values related to ecology (23/ 767, 3% and 44/1238, 3.5%, respectively). With respect to human rights, patients and public participants considered digital pills to be a policing tool (112/767, 14.6% and 143/1238, 11.6%, respectively) and worried about the associated intrusiveness (66/767, 8.6% and 150/1238, 12.1%, respectively). Regarding medical care, patients and public participants mostly reported concerns with confidentiality (44/767, 5.7% and 95/1238, 7.7%, respectively) and disempowerment of patients (44/767, 5.7% and 44/1238, 3.6%, respectively). To prevent the unethical use of digital pills, they requested further investigation of the associated harms and benefits (50/ 767 patients, 6.5%; 96/ 1238 public participants, 7.8%) and that the use of these pills be restricted to clinical research (9/767 patients, 1.2%; 11/1238 public participants, 0.9%).

In total, 29/246 (11.8%) health care professionals considered digital pills to be part of technological progress that aligned with their values; they mostly worried about policing (90/246, 36.6%) and intrusiveness (62/246, 25.2%).

Participants were afraid that digital pills might cause a breakdown in trust between patients and doctors (36/767 patients, 4.7%; 39/1238 public participants, 3.1%; 147/246 health care professionals, 59.8%), which is seen as the cornerstone of the patient–doctor relationship.

I don't like being kept under surveillance. There is a relationship of trust between my GP, my psychiatrist and me. This would undermine that trust because I would be policed.

Patient, woman, 51 years old

Ethically it's very questionable, it makes it look like we're ‘spying on’/‘policing’ the patient. What about the patient's freedom? Could such a device really improve patient compliance? A good motivational interview seems more relevant. (It is better to act upstream = prevention and health promotion, therapeutic education, rather than downstream = bad compliance to be ‘reprimanded’) Not to mention that this would put the patient at odds vis-à-vis the doctor in case of poor compliance... And in this case, what about the doctor-patient trust relationship?

General practitioner, woman, 29 years old

### Perceived Opportunity

Participants identified advantages (6/98 codes, 6%), disadvantages (8/98 codes, 8%), and specific target populations for whom they considered digital pills would be beneficial (16/98 codes, 16%).

A total of 135/767 (17.6%) patients and 200/1238 (16.2%) public participants had an overall positive perception of digital pills, and 213/767 (27.8%) patients and 408/1238 (33%) public participants had an overall negative perception of digital pills. Patients and public participants mostly reported real-time data (70/767, 9.1% and 181/1238, 14.6%, respectively) as an advantage and the addition of another connected device in an already saturated environment (74/767, 9.6% and 86/1238, 6.9%, respectively) as a disadvantage. Regarding potential target populations, some highlighted that digital pills were not suitable for themselves (107/767 patients, 14%; 106/1238 public participants, 8.6%) and reported mostly people with cognitive impairment (87/767 patients, 11.3%; 87/1238 public participants, 7%) or older people (48/767 patients, 6.3%; 81/1238 public participants, 6.5%) to be suitable candidates.

Health care professionals pointed out the precision of digital pills (15/246, 6.1%) and the ease to discuss treatment (eg, tolerance, benefits, adherence) (11/246, 4.5%) as advantages and the cost (41/246, 16.7%) as a disadvantage. They identified people with cognitive impairment (29/246, 11.8%) and chronic conditions (19/246, 7.8%) as the main target population.

Participants identified people living under judicial constraint as a potential target population for digital pills (1/767 patients, 0.9%; 6/1238 public participants, 0.5%; 2/246 health care professionals, 4.6%); these people were depicted as dangerous, unwilling to undergo treatment, and already under surveillance and deprived of liberty, which—according to participants—would make the ethical regimen more flexible for them:

The person will take his treatment because he is compelled to do so by a court order. He is either a delinquent or a criminal. For someone who has not broken the law, there is no reason to proceed in this way.

Public participant, man, 39 years old

I think it can be a good idea for people who are sick (Alzheimer's for example) and forget to take their medication. Or for more complex cases such as people who are dangerous to society and the doctor needs to be sure that the person's treatment has been taken.

Public participant, woman, 43 years old

### Affective Attitude

We identified 8 affective attitudes toward digital pills: enthusiasm (100/767 patients, 13%; 146/1238 public participants, 11.8%; 20/246 health care professionals, 8.1%), curiosity (141/767 patients, 18.4%; 170/1238 public participants, 13.7%; 31/246 health care professionals, 12.6%), balanced attitude (70/767 patients, 9.1%; 111/1238 public participants, 9%; 41/246 health care professionals, 16.7%), unease (32/767 patients, 4.2%; 70/1238 public participants, 5.7%; 7/246 health care professionals, 2.9%), skepticism (114/767 patients, 14.9%; 168/1238 public participants, 13.6%; 31/246 health care professionals, 5.3%); fear (56/767 patients, 7.3%; 175/1238 public participants, 14.1%; 13/246 health care professionals, 5.3%), anger (178/767 patients, 23.2%; 255/1238 public participants, 20.6%; 108/246 health care professionals, 43.9%), and disgust (7/767 patients, 0.9%; 4/1238 public participants, 0.3%; 1/246 health care professional, 0.4%). Because of lack of clarity and risk of overinterpretation, we could not code the affective attitude of 69/767 (9%) patients, 139/1238 (11.2%) public participants, and 12/246 health care professionals (4.9%). Compared to public participants, patients had more positive reactions (316/1238, 25.5% vs 241/767, 31.4%) and less negative reactions (672/1238, 54.3% vs 387/ 767, 50.5%). Compared to patients and public participants, health care professionals expressed more negative attitudes (142/246, 57.7%) and less positive attitudes (51/246, 20.7%).

### Willingness to Take Digital Pills

**Figure 3:**
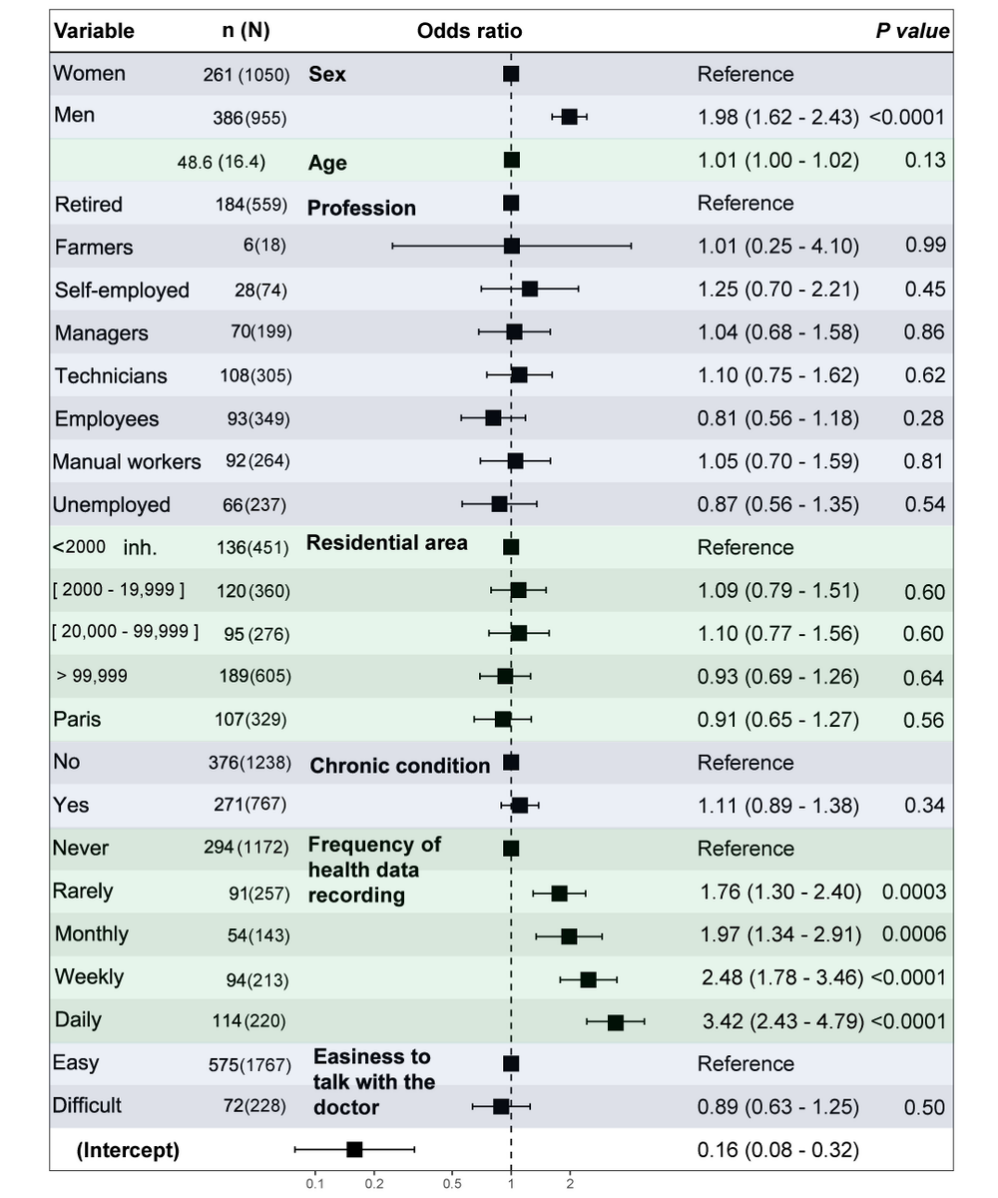
Determinants of the willingness to take connected drugs among the representative sample of patients and public participants (N=2005). The second column present the number of people willing to take connected drugs (n) over the total number in the patient and public sample (N), except fot the variable "Age", presenting the mean and standard deviation (sd) of people willing to take connected drugs. inh.: inhabitants.

In the representative sample, 647/2005 (32.3%) participants declared that they would use a digital pill: 271/767 (35.3%) patients, 376/1238 (30.4%) public participants, and 39/246 (15.8%) health care professionals. After adjustment, willingness to take digital pills by patients and public participants was significantly associated with male sex (OR 1.98, 95% CI 1.62-2.43) and integration of currently used connected devices to monitor health daily (OR 3.42, 95% CI 2.43-4.79) or rarely (OR 1.76, 95% CI 1.30-2.40), with a dose–response relationship (Figure 3). Complete analysis of other characteristics associated with the willingness to take a digital pill is presented with crude and adjusted ORs and 95% CIs in Multimedia Appendix 10. The forest plot (Figure 3) presents the ORs with their 95% CIs and the *P* value obtained with the logistic regression analysis of the association of the willingness to take connected drugs by sex, age, profession, population density of the residential area (“residential area”), chronic condition, frequency of health data recording with a connected device, and ease to talk about treatments with the doctor. The plot shows the number of participants per category (n) willing to take a connected drug among the total number of participants in the given category (N). The corrected threshold for statistical significance is *P*=.003. For the continuous variable “age,” we present the mean age of the whole sample and the standard deviation.

## Discussion

### Principal Findings

Overall, two-third of patients and public participants in this study refused to take digital pills. Their arguments were grounded in the fear of possible serious clinical and ethical harms. Health care professionals largely refused (177/246, 72%) to take digital pills citing disagreement with their professional deontology and ethics. Participants who accepted digital pills had positive a priori for technology considered as progress: people agreed to take it in the framework of research for a limited investigation time to help medical progress.

Participants who refused to take connected drugs, reported that these would be useful for other people, such as individuals with cognitive impairment, individuals with chronic conditions or mental disorders, and those incarcerated, highlighting the view that potential clinical or ethical harms would be acceptable for these already vulnerable people. Given the condition of the designated populations, the intervention may be stigmatizing and may increase vulnerability.

To our knowledge, this study is the first to explore the acceptability of connected drugs in line with the recommendation that health technology assessment take into account patient and public perspectives [18]. We used a representative sample of the French population, thus allowing for generalizable results in the subsamples of patients and the public. In France, medication for chronic conditions is fully reimbursed, so when participants answered the questionnaire, they were not prematurely restricted by individual economic concerns but could detail their preferences. To limit the risk of capturing only a “hot reaction” and to mimic the process of a rational and balanced deliberation, we used 5 open-ended questions and asked the close-ended question about willingness to use connected drugs at the end of the survey. Only 16.7% of patients and public participants found no cons and 31% found no pros.

### Limitations

The first limitation is the restriction to only 1 country as a consequence of the choice to recruit a representative sample. Because culture and health care system may affect the acceptability of digital pills, further studies are required in different settings. 

A common limitation of using an online survey is the risk of selection bias because of the need for internet access. However, the target population for the digital pill must have access to smartphones and the internet. While the use of an online survey may have overestimated the acceptability of digital pills in the general population, it may not have done so in the target population. Moreover, to limit potential selection bias due to reading level, we created the questionnaire during a qualitative preliminary study and tested it for clarity.

We could have increased the reliability of the results by verifying the final codes and themes with the participants, but we minimized the risk of misinterpretation by limiting the condensation of codes to synonyms only, halting the inductive interpretation at a very basic level—which led to 98 codes in the final results—and using the participants’ wording. 

Another limitation is that the sample of health care professionals was not representative of the larger population of health care professionals.

Finally, we only investigated the prospective acceptability because digital pills were not available in France at the time of our study. Studies about concurrent acceptability (while taking connected drugs) and retrospective acceptability (after taking connected drugs) may be complementary and lead to different results.

### Comparison With Prior Work

Our results are consistent with the concurrent and retrospective acceptability discussed in 18 observational studies and trials of digital pills, which involved small samples of 5 to 129 patients [26]. Moreover, some participants who refused digital pills for themselves found them useful for other people considered vulnerable (eg, people with cognitive or mental disorders or those incarcerated), which would lead to a risk of stigmatizing the use of digital pills.

This study added further ethical concerns to the well-known topics raised in the scientific literature (eg, consent, confidentiality, privacy) [12,26-30]. This shows the substantial importance of including patient and public perspectives in health technology assessment, as is already recommended but marginally done, with the risk of neglecting social, ethical, and political aspects of these technologies that are critical determinants of treatment effectiveness in real life settings [18]. A further strength is the use of a representative sample, which allows for generalizable results.

This survey contributes to advanced scientific and clinical reflections about medication adherence. As underlined by participants, digital pills seem to be a naïve solution considering the complexity of what determines medication adherence and nonadherence [12,31,32]. Digital pills appear to be burdensome and expensive reminders with an apparent contradiction—whether unintentional or intentional—between their complexity and the ability of those needing support for adherence. Particularly in the case of intentional nonadherence, digital pills would be either useless or efficient at the cost of intrusiveness, policing, and negative effects on the patient–doctor relationship.

Finally, digital pills could be useful to assess treatment efficacy in explanatory trials, but there are 2 prerequisites for extending their use in clinical practice. First, digital pills should be evaluated as a complex intervention according to specific standards and not as if they are a mere change in the form of drug administration [17,33]. Second, there is urgent need to develop an ethical and legal framework to ensure the safe and ethical collection and use of health data through a patient-centered approach [12,27,28].

### Conclusion

Our results suggest that patients, the public, and health care professionals view connected drugs not as a promising new medical device to better monitor medication adherence but as a complex intervention with a possible impact on patient–doctor relationship and patient autonomy. The participants also raised additional concerns about burden of treatment, cost-effectiveness, and privacy, which need to be addressed in further investigations. Future studies should take into account the views of all stakeholders, ie, patients, potential prescribers, as well as health regulatory authorities and researchers, to ensure the safe and ethical collection and use of health data through a patient-centered approach.

## Appendix

Multimedia Appendix 1 Determinants of the willingness to take digital pills.

Multimedia Appendix 2 Questionnaire for healthcare professionals.

Multimedia Appendix 3 Calculation of the weight for the representative sample of the French general population (patients and public).

Multimedia Appendix 4 Methods for the online recruitment of healthcare professionals.

Multimedia Appendix 5 Preliminary qualitative study.

Multimedia Appendix 6 Chronic conditions of patients.

Multimedia Appendix 7 Description of healthcare professionals.

Multimedia Appendix 8 Data saturation.

Multimedia Appendix 9 Acceptability of digital pills.

Multimedia Appendix 10 Determinants of the willingness to take digital pills.

## Abbreviations

OR: odds ratio
