# Supplementary material for: Acceptability of and Willingness to Take Digital Pills by Patients, the Public, and Health Care Professionals: Qualitative Content Analysis of a Large Online Survey
Source: J Med Internet Res. 2022 Feb 18;24(2):e25597. doi: 10.2196/25597 (PMC8900921; doi:10.2196/25597)
Supplement: Multimedia Appendix 2 [file jmir_v24i2e25597_app2.docx]

# Multimedia Appendix 2: Questionnaire for healthcare professionals

**You are**

A woman

A man

Another answer:

**Your age**

**What is your profession?**

Physician

Nurse

Pharmacist

Midwife

Another answer:

**[if PHYSICIAN] What is your specialization?**

ALLERGOLOGY and CLINICAL IMMUNOLOGY

PATHOLOGICAL ANATOMY and CYTOLOGY

ANDROLOGY

ANESTHESIA REANIMATION

HORMONAL and METABOLIC BIOCHEMISTRY

BIOLOGY of INFECTIOUS AGENTS

MEDICINAL BIOLOGY

MOLECULAR BIOLOGY

CARDIOLOGY and VASCULAR DISEASES

FACIAL and NECK SURGERY

GENERAL SURGERY

MAXILLOFACIAL SURGERY

ORTHOPEDIC SURGERY and TRAUMATOLOGY

PEDIATRIC SURGERY

PLASTIC AND RECONSTRUCTION SURGERY

THORACIC and CARDIO-VASCULAR SURGERY

UROLOGICAL SURGERY

VASCULAR SURGERY

VISCERAL SURGERY

DENTAL SURGERY – DENTISTRY

HUMAN CYTOGENETICS

DERMATOLOGY and VENEROLOGY

ENDOCRINOLOGY, DIABETES and METABOLIC DISEASES

FETOPATHOLOGY

GASTRO - ENTEROLOGY and HEPATOLOGY

MEDICAL GENETICS

GERIATRY

MEDICAL GYNECOLOGY

OBSTETRICAL GYNECOLOGY

BIOLOGICAL HEMATOLOGY

BLOOD HEMATOLOGY and BLOOD DISEASE

ONCOLOGIC HEMATOLOGY

TRANSFUSION HEMOBIOLOGY

HYGIENE

IMMUNOLOGY and IMMUNOPATHOLOGY

MEDICAL INFORMATION (SUN)

PAIN MEDICINE and PALLIATIVE MEDICINE

REPRODUCTION MEDICINE

SPORT MEDICINE

WORK MEDICINE

EMERGENCY MEDICINE – SAMU

GENERAL MEDICINE

INTERNAL MEDICINE

NUCLEAR MEDICINE

PHYSICAL and READAPTATION MEDICINE

VASCULAR MEDICINE

NEONATOLOGY

NEPHROLOGY

NEUROSURGERY

NEUROLOGY

NUTRITION

O.R.L. and CERVICO-FACIAL SURGERY

MEDICAL ONCOLOGY

RADIOTHERAPY ONCOLOGY

OPHTALMOLOGIE

INFECTIOUS and TROPICAL, CLINICAL AND ORGANIC PATHOLOGY

PEDIATRY

PHARMACOCINETICS and METABOLISM of MEDICINALS

CLINICAL PHARMACOLOGY and THERAPEUTIC EVALUATION

PNEUMOLOGY

PSYCHIATRY

CHILD and TEEN PSYCHIATRY

RADIODIAGNOSTIC and MEDICAL IMAGERY

RADIOPHARMACY and RADIOBIOLOGY

MEDICAL REANIMATION

RHUMATOLOGY

PUBLIC HEALTH and SOCIAL MEDICINE

STOMATOLOGY

BIOLOGICAL TOXICOLOGY

**How many years have you been in this profession? (For doctors and pharmacists, please count your years of internship)**

Certain connected objects such as mobile phones, watches or other sensors can be used to record health parameters such as heartbeat, sleep rhythm, number of steps taken, number of calories consumed, etc.

**Personally, do you consult your health parameters as recorded by connected objects?**

Yes, every day

Yes, several times a week

Yes, several times a month

Yes, rarely

No, never

I do not wish to answer

**Do you have a chronic illness (or more than one)? An illness is chronic if it requires regular treatment and/or follow-up (consultation, medication, rehabilitation, special diet, etc.) for at least six months.**

Yes

No

I do not know

I do not wish to answer

IF yes: which one(s)

**[if YES] Which one(s) ?**

**[if YES] Approximately how long have you been living with a chronic illness (duration of first chronic illness diagnosed)**

*If your illness was diagnosed less than one month ago, enter one month.*

In years

In months

**Have you had a treatment prescribed by a doctor to be taken regularly for more than a month?**

This could be, for example, tablets, eye drops, injections, implants or inhalers prescribed by a doctor (excluding contraceptives, excluding pregnancy)

Yes, I have been prescribed one (or more) medication(s) to take every day

Yes, I have been prescribed one (or more) medication(s) to be taken once or several times a month. For example, once a day, or once every week, or every other day.

No, I have not been prescribed regular treatment

I do not wish to answer

**[if YES] How long have you been taking a regular treatment?**

In years

In months

**[if YES] Over the last month, have you ever failed to take this treatment (forgot, taking a break, holidays, weekends, not wanting to take it, etc.)?**

No, I have never forgotten it

Yes, it has happened to me less than once a week

Yes, it has happened to me several times a week

Yes, it happens to me almost every day

I never started the treatment prescribed on the prescription

I prefer not to answer that question

For those who answered no, or I do not wish to answer question 5

**[if NO] When you have been prescribed treatment for a specific problem, do you ever cut it short or reduce the doses on your own initiative? For example, many people stop taking their antibiotic one or two days before the prescription runs out.**

Yes, this often happens to me

Yes, this sometimes happens to me

Yes, this rarely happens to me

No, this never happens to me

**Do you feel free to discuss your treatment with your doctor, including voicing your reluctance to take it, speaking about potential side effects, etc.?**

Yes, most of the time I can discuss these topics with my doctor

No, most of the time, I dare not talk about it

I prefer not to answer that question

**During the year 2019, how many times did you consult a doctor (general practitioner, specialist, emergencies)? This question may tax your memory, it is normal not to remember the exact number of consultations. It is sufficient to indicate an order of magnitude.**

More than 10 times

Between 5 and 10 times

Fewer than 5 times

I did not see a doctor during that year

I do not wish to answer that question

**Do you assess patient compliance?**

Systematically, i.e. for each patient under treatment and at each consultation

Selectively, i.e. for certain patients or situations

Rarely

**[if SELECTIVELY] Please specify which ones**

**[if RARELY] Please indicate why (difficult subject to address, I don't know what to do in case of bad compliance, I don't think about it, etc )**

**Here is a type of tablet that should soon be marketed in France. We are showing it to you to obtain your opinion and your thoughts on this subject through four open questions.**

IMAGE (to keep on the same page)

Open-ended questions

1. **What do you think of this device (transmitter + Bluetooth patch + smartphone app + access to data)? We want to know your immediate reaction: try to write down all the ideas that came to you when you first saw this device.**

**There is no right or wrong answer. Moreover, you can make your answer as long as you wish.**

1. **What positive aspects could you envisage in this device (transmitter + Bluetooth patch + smartphone app + access to data)? If you do not see any or have no ideas, please write “None”.**

**For example, in what situation(s)/for whom could it be useful, what kind of benefit could it bring? There is no right or wrong answer. Moreover, you can make your answer as long as you wish.**

1. **What negative aspects could you envisage in this device (transmitter + Bluetooth patch + smartphone app + access to data)? If you do not see any or have no ideas, please write “None”.**

**For example, what drawbacks or risks do you foresee? There is no right or wrong answer. Moreover, you can make your answer as long as you wish.**

1. **If your doctor suggested a treatment using this device (transmitter + Bluetooth patch + smartphone app + access to data), what would your reaction be? What would you think of his approach?**

**There is no right or wrong answer. Moreover, you can make your answer as long as you wish.**

1. **The manufacturer says that this device will allow for greater consistency between the prescription and the actual taking of medications. In your opinion, why would a person take their treatment more consistently if they were equipped with this device?**

**There is no right or wrong answer. Moreover, you can make your answer as long as you wish.**

**Would you agree to use this device for yourself?**

Yes

No

I do not wish to answer

**Why?**

**Please describe your "tips" for assessing patients’ adherence, if you have any?**

**There is no right or wrong answer. Furthermore, you can make an answer as long as you wish.**
