# Supplementary material for: Acceptability of and Willingness to Take Digital Pills by Patients, the Public, and Health Care Professionals: Qualitative Content Analysis of a Large Online Survey
Source: J Med Internet Res. 2022 Feb 18;24(2):e25597. doi: 10.2196/25597 (PMC8900921; doi:10.2196/25597)
Supplement: Multimedia Appendix 3 [file jmir_v24i2e25597_app3.docx]

# Multimedia Appendix 3: Calculation of the weight for the representative sample of the French general population (patients and public)

| **Characteristics – n (%)** | **Raw value** | **Weight** | **Weighted value ^a^** |
| --- | --- | --- | --- |
| **Woman X Age** |  |  |  |
| Age [18-24] years  Age [25-34] years  Age [35-44] years  Age [45-59] years  Age > 59 years | 86 (4.3)  182 (9.1)  176 (8.8)  221 (11.0)  381 (19.0) | 1.187  0.884  0.973  1.204  0.918 | 102 (5.1)  161 (8.0)  171 (8.5)  266 (13.3)  350 (17.4) |
| **Man X Age** |  |  |  |
| Age [18-24] years  Age [25-34] years  Age [35-44] years  Age [45-59] years  Age > 59 years | 43 (2.1)  113 (5.6)  165 (8.2)  319 (15.9)  319 (15.9) | 2.383  1.356  1.010  0.793  0.877 | 102 (5.1)  153 (7.6)  167 (8.3)  253 (12.6)  280 (14.0) |
| **French area** |  |  |  |
| Paris agglomeration  Northwest  Northeast  Southwest  Southeast | 373 (18.6)  458 (22.8)  450 (22.4)  222 (11.1)  502 (25.0) | 0.995  0.999  0.995  1.022  1.000 | 371 (18.5)  458 (22.8)  448 (22.3)  227 (11.3)  502 (25.0) |
| **Population density of the place of residence (inhabitants)** |  |  |  |
| Rural city (< 2000)  [2000-19,999]  [20,000-99,999]  ≥ 100,000  Paris agglomeration | 425 (21.2)  360 (18.0)  276 (13.8)  613 (30.6)  331 (16.5) | 1.061  0.969  0.981  0.988  0.994 | 451 (22.5)  349 (17.4)  271 (13.5)  605 (30.2)  329 (16.4) |
| **Socio-professional category** |  |  |  |
| Farmers | 13 (0.6) | 1.388 | 18 (0.9) |
| Self-employed professional workers | 72 (3.6) | 1.030 | 74 (3.7) |
| Senior managers | 201 (10.0) | 0.988 | 198 (9.9) |
| Technicians and associate professionals | 304 (15.2) | 1.002 | 305 (15.2) |
| Junior non-manual workers | 403 (20.1) | 0.866 | 349 (17.4) |
| Manual workers | 219 (10.9) | 1.208 | 265 (13.2) |
| Retired people | 613 (30.6) | 0.913 | 559 (27.9) |
| Unemployed | 180 (9.0 | 1.314 | 237 (11.8) |

**^a^** Weights have been calculated with the rim weighting method (raking) to correspond to the INSEE 2015
