# Supplementary material for: Acceptability of and Willingness to Take Digital Pills by Patients, the Public, and Health Care Professionals: Qualitative Content Analysis of a Large Online Survey
Source: J Med Internet Res. 2022 Feb 18;24(2):e25597. doi: 10.2196/25597 (PMC8900921; doi:10.2196/25597)
Supplement: Multimedia Appendix 4 [file jmir_v24i2e25597_app4.docx]

# Multimedia Appendix 4: Methods for the online recruitment of healthcare professionals

We recruited professionals by:

1. Advertisements on social networks:
   1. Facebook groups of medical interns (psychiatry, general medicine, etc.)
   2. Twitter
   3. LinkedIn
2. Mailings to various specialty associations (interns, seniors, etc.)
   1. Association of Parisian psychiatric interns (PEPS)
   2. Federation of psychiatric interns in France (AFFEP)
   3. Association of interns in anesthesia-reanimation (AJAR)
   4. Association of general medical interns (ISNAR-MG)
   5. Association of Parisian general medical interns (SPR-IMG)
   6. National Federation of Pharmacy and Medical Biology Residents' Unions (FNSIP-BM)
   7. Union of pharmacy and medical biology interns in Ile-de-France hospitals (SIPHIF)
   8. National Internship Union (ISNI)
   9. National youth surgery council (CNJC)
   10. Association of Assistants and Interns in Neurology (ANAINF)
   11. National union of interns and young radiologists (UNIR)
   12. Association of Young Emergency Physicians (AJMU)
   13. National Union of Interns in Endocrinology and Diabetology (UNITED)
   14. National student midwifery association (ANESF)
   15. Midwives association (APSF)
   16. National Union of Liberal Nurses (SNIIL)
   17. French National Association of Nurses and Nursing Graduates and Students (ANFIIDE)
   18. Association of young psychiatrics and young addictologists (AJPJA)
   19. Intersyndicate of Hospital Practitioners (INPH)
   20. Confederation of French trade unions (CSMF)
   21. French Cardiology Federation (FFC)

**Example of an advertisement for Facebook groups:**

Digital pills will be soon available. It is an ingestion sensor embedded in a tablet to know in real time the patients' medication intake. What is your opinion? What will be the impact on the doctor-patient relationship?

We are conducting a scientific study to find out what prescribers think about these particular drugs.

By filling out this questionnaire, which takes less than 10 minutes, you will contribute to the evaluation of these new therapeutics.

**Example of an advertisement for mails:**

Dear colleagues,

Digital pills will be available soon. These are ingestion sensors embedded in the tablets of conventional medicines to monitor patient compliance.

We are currently conducting a scientific study to find out your views on these devices. We invite you to fill out a questionnaire in less than 10 minutes by logging in at the following link. Your doctor's opinion is important for the evaluation of this medical device.

**Example of advertisements for Twitter:**

Type 1: Healthcare professional: drugs connected, opportunity or danger? Give your opinion in this scientific study. #digital pills

Type 2: Monitoring compliance with connected medications, what do you think? Have your say in this scientific study. #digital pills

Type 3: A drug connected to know patient compliance? Healthcare professionals, give your opinion in this scientific study. #digital pills
