# Supplementary material for: Acceptability of and Willingness to Take Digital Pills by Patients, the Public, and Health Care Professionals: Qualitative Content Analysis of a Large Online Survey
Source: J Med Internet Res. 2022 Feb 18;24(2):e25597. doi: 10.2196/25597 (PMC8900921; doi:10.2196/25597)
Supplement: Multimedia Appendix 5 [file jmir_v24i2e25597_app5.docx]

# Multimedia Appendix 5: Preliminary qualitative study

**Objective**

1) to observe the reaction, identify the perception, and understand the perspectives of people from the general population, patients with chronic conditions and healthcare professionals when being told about digital pills.

2) to develop the questions to be used in the online survey (phrasing, formatting, content) for each group

**Method**

A qualitative study based on semi-directed interviews was conducted and qualitative thematic analysis was conducted by a clinical researcher trained in social science (AC).

*Population*

- Patient: any adult (≥ 18 years old) with ongoing chronic condition and long-term treatment.
- HCP: professional who prescribe and monitor the treatments of patients with chronic condition
- General population: adult ≥ 18 years old without chronic condition

Participants were volunteers. They provided oral consent to participate and to be audio-recorded before the beginning of the interview. All interviews were full anonymous during transcribing (no name, no first name, no birthdates asked).

*Sampling method*

**Purposeful sampling**: maximum variation sampling (and phenomenal variation sampling for the patients) (Sandelowski 1995; Coyne 1997) (see Fig. 2). We made the assumption that the following variables influence the diversity of expectations for each group.

- - For patients:
    - Maximum variation sampling with sex, socioeconomic level defined by educational level (high school or less/more) and income (< 13 788/year (or ≥ 1149/month minimum wage)
    - for the phenomenal variation: diversity of the chronic condition
  - For HCPs: occupation (psychiatrists, other medical specialties)
  - For the public: sex, age, educational level (high school or less/more) and income (< 13 788/year (or ≥ 1149/month minimum wage).

**Number of participants**:

As recommended by qualitative research, the number of participants should be about 20. (Creswell 2012; Denzin et Lincoln 2011; Sandelowski 1995; Dworkin 2012) We chose to study at least 8 patients, 4 informal caregivers and 6 HCPs to be informative enough while feasible.

**Recruitment**

Patients

Patients were recruited by a GP in a private consultation in Paris (SS). The GP in charge of the patient proposed that the patient participate in the study. Patients who agreed were then contacted by the principal investigator who explained the purpose and modalities of the study.

Healthcare professionals

HCPs were recruited in hospitals (Centre Hospitalier Sainte Anne, Paris and in Hôpital Cochin, Paris).

The Public

Young people were recruited in university or professional schools of the Paris region and in two towns in France, by word of mouth

***Data collection***

- For the investigator: writing about her own reaction, perception and opinion about the digital pills and also about preconceptions of patients, public, and HCP anticipated reaction, perception and opinion. This elaboration helps the investigator set aside her own preconceptions and experience (“bracketing”/“epoche” (Gearing 2004)).
- Face-to-face semi-structured interviews in French about the perception and the opinion toward the digital pills. The investigator encouraged the interviewees to speak about their own experience as a patient, HCP or general population of disease, drugs benefits and harms, adherence. The digital pill was presented to each participant with the help of a picture, written and oral explanation. Interviews were registered with the consent of the interviewees. Semi-structured interviews were based on the following framework, which could be modified across interviews.

| **Framework for the face-to-face semi-structured in-depth interview:**  **Introduction of the interviewer**  **Introduction of the research**  **Informed consent about the research and the registration of the interview (written information sheet, oral consent)**  **Questions about the experience of chronic condition and long-term treatment if relevant and/or short-term condition and treatment**  **Questions about the experience of adherence to short term and long-term treatment**  **Questions about the relationship with the family doctor and with other doctors**  **Presentation of the digital pill with the help of the picture and observation of the immediate reaction**  **Elicitation of the perception of the digital pill (immediate and delayed reactions throughout the interview)**  **The understanding of the digital pills**  **Questions about the potential positive and negative characteristics of the digital pill.**  **Questions about the acceptability of the drug (burden, ethical conflict, perceived effectiveness, self-efficacy, willingness to test, etc.)**  **Debriefing of the interview**  **Screening questions according to status:**   - for patients: sex, age, socioeconomic level, chronic condition, long term treatment - for HCPs: profession, sex, age, experience (years of activity) - for the public: sex, age, socioeconomic level, chronic condition/long-term treatment |
| --- |

**Fig. 1: Framework for the face-to-face semi-structured in-depth interview.**

*Analysis*

- - Inductive thematic analysis after reading and rereading the interviews. Open-coding by one researcher (AC) in her mother tongue
  - Identification of the questions and wording that were able to retrieve rich and accurate responses

*Results*

Description of the population

In May and June 2019, AC conducted 12 face-to-face interviews with French participants (5 patients, 3 HCPs and 5 from the public).

Patients included:

- one man, 52 years old, with low socioeconomic level with diabetes, chronic renal impairment and hypertension, under multiple long-term treatment for 10 years.
- one woman, age 62 years old, with very low socioeconomic level, HIV and long-term treatment for 12 years
- one woman, 66 years old, with high socioeconomic level, multiple sclerosis and long-term treatment for 25 years
- one woman 28 years old, with intermediate socioeconomic level, bipolar disorder and long-term treatment for 1.5 years
- one woman 58 years old with intermediate socioeconomic level and Hashimoto thyroiditis, under long-term treatment for 20 years

HCPs included:

- One women age 43 years old, psychiatrist in a psychiatric hospital
- One women age 35 years old, infectious disease specialist in a general hospital
- One man age 28 years old, internal medicine resident in a general hospital

Public participants included:

- One man, 20 years old, high socioeconomic level, third-year student in public policies
- One women 23 years old, low socioeconomic level childcare professional
- One man 34 years old, high socioeconomic level working as a trader in an insurance company
- One man 58 years old, intermediate socioeconomic level, seller
- One man 54 years old, high socioeconomic level, biology teacher

Thematic Analysis

Results not reported here.

Reactions to the questions and development of the questions

No participants were aware of digital pills, and all were surprised by learning about it.

The final questions developed with this qualitative study and used in the online survey are reported in box 1 of the article.

**References**

Boynton, Petra M, et Trisha Greenhalgh. 2004. « Selecting, designing, and developing your questionnaire ». *BMJ : British Medical Journal* 328 (7451): 1312‑15.

Burns, Karen E.A., et Michelle E. Kho. 2015. « How to assess a survey report: a guide for readers and peer reviewers ». *CMAJ : Canadian Medical Association Journal* 187 (6): E198‑205. https://doi.org/10.1503/cmaj.140545.

Coyne, I. T. 1997. « Sampling in Qualitative Research. Purposeful and Theoretical Sampling; Merging or Clear Boundaries? » *Journal of Advanced Nursing* 26 (3): 623‑30.

Creswell, John W. 2012. *Qualitative Inquiry and Research Design: Choosing Among Five Approaches*. Third Edition edition. Los Angeles: SAGE Publications, Inc.

Denzin, Norman K., et Yvonna S. Lincoln, éd. 2011. *The SAGE Handbook of Qualitative Research*. Fourth Edition edition. Thousand Oaks: SAGE Publications, Inc.

Dworkin, Shari L. 2012. « Sample Size Policy for Qualitative Studies Using In-Depth Interviews ». *Archives of Genderual Behavior* 41 (6): 1319‑20. https://doi.org/10.1007/s10508-012-0016-6.

Foddy, William. 1994. *Constructing Questions for Interviews and Questionnaires: Theory and Practice in Social Research*. Cambridge University Press.

Gearing, Robin Edward. 2004. « Bracketing in Research: A Typology ». *Qualitative Health Research* 14 (10): 1429‑52. https://doi.org/10.1177/1049732304270394.

Sandelowski, M. 1995. « Sample Size in Qualitative Research ». *Research in Nursing & Health* 18 (2): 179‑83.
