# Supplementary material for: Acceptability of and Willingness to Take Digital Pills by Patients, the Public, and Health Care Professionals: Qualitative Content Analysis of a Large Online Survey
Source: J Med Internet Res. 2022 Feb 18;24(2):e25597. doi: 10.2196/25597 (PMC8900921; doi:10.2196/25597)
Supplement: Multimedia Appendix 6 [file jmir_v24i2e25597_app6.docx]

# Multimedia Appendix 6: Chronic conditions of patients

| **Chronic conditions of the patients (**N=767) |  |  |
| --- | --- | --- |
| **Duration of the chronic condition – mean (SD), years** | 13.6 (11.7) |  |
| **Duration of the long-term treatment – mean (SD), years** | 10.2 (9.9) |  |
|  |  |  |
| **Names of the chronic conditions – n, %** |  |  |
| Hypertension | 169 | 22.0 |
| Diabetes | 135 | 17.6 |
| Chronic pain (e.g., arthrosis, low back pain) | 81 | 10.6 |
| Thyroid disease | 70 | 9.1 |
| Heart disease (e.g., arrythmia, heart failure) | 63 | 8.2 |
| Asthma | 62 | 8.1 |
| Dyslipidemia | 56 | 7.3 |
| Depression and anxiety | 38 | 5.0 |
| Coronary heart disease | 29 | 3.8 |
| Cancer | 28 | 3.7 |
| Allergia | 27 | 3.5 |
| Inflammatory disease (e.g., Horton. rheumatoid arthriris) | 25 | 3.3 |
| Respiratory disorder (COPD. bronchectiasis) | 23 | 3.0 |
| Digestive disorder (reflux. gastric ulcer) | 18 | 2.3 |
| Sleep apnea | 14 | 1.8 |
| Migraine headache | 13 | 1.7 |
| Stroke | 12 | 1.6 |
| Chronic inflammatory bowel disease | 12 | 1.6 |
| Bipolar disorder | 11 | 1.4 |
| Autoimmune disease | 11 | 1.4 |
| Ear nose throat disease | 10 | 1.3 |
| Other infections | 10 | 1.3 |
| Coagulation disorder | 9 | 1.2 |
| HIV | 9 | 1.2 |
| Multiple sclerosis | 9 | 1.2 |
| Glaucoma | 9 | 1.2 |
| Epilepsy | 7 | 0.9 |
| Osteoporosis | 6 | 0.8 |
| Psoriasis | 6 | 0.8 |
| Prostate disease | 6 | 0.8 |
| Renal disease | 5 | 0.7 |
| Verneuil disease | 5 | 0.7 |
| Paralysia | 5 | 0.7 |
| Obesity | 5 | 0.7 |
| Other dermatological disorder | 5 | 0.7 |
| Irritable bowel syndrome | 4 | 0.5 |
| Eating disorder | 4 | 0.5 |
| Anemia | 4 | 0.5 |
| Eczema | 3 | 0.4 |
| Endometriosis | 3 | 0.4 |
| Parkinson disease | 3 | 0.4 |
| Insomnia | 3 | 0.4 |
| Ocular hypertension | 3 | 0.4 |
| Inherited condition | 3 | 0.4 |
| Hydrocephalus | 3 | 0.4 |
| Transplantation | 2 | 0.3 |
| Non-informative | 2 | 0.3 |
| Dizziness | 1 | 0.1 |
| Schizophrenia | 1 | 0.1 |
| AMD | 1 | 0.1 |
| PCOS | 1 | 0.1 |

COPD, chronic obstructive pulmonary disease; AMD, age-related macular degeneration; PCOS, polycystic ovary syndrome
