# Supplementary material for: Acceptability of and Willingness to Take Digital Pills by Patients, the Public, and Health Care Professionals: Qualitative Content Analysis of a Large Online Survey
Source: J Med Internet Res. 2022 Feb 18;24(2):e25597. doi: 10.2196/25597 (PMC8900921; doi:10.2196/25597)
Supplement: Multimedia Appendix 7 [file jmir_v24i2e25597_app7.docx]

# Multimedia Appendix 7: Description of healthcare professionals

| **Question** | **All HCPs**  **N=246** | **With a chronic disease N=28 (15.5%)** | **Without a chronic disease**  **N=218 (88.6%)** |
| --- | --- | --- | --- |
|  |  |  |  |
| **Self-reported chronic condition (chronic disorder) – n (%)*** |  |  |  |
| Hypertension | 7 (2.8) | 6 (21.4) | 1 (0.5) |
| Asthma | 5 (2.0) | 5 (17.9) | 0 (0) |
| Thyroid disorders | 4 (1.6) | 3 (10.7) | 1 (0.5) |
| Mental disorders | 6 (2.4) | 6 (21.4) | 0 (0) |
| Cardiovascular disorders (excluding hypertension) | 3 (1.2) | 1 (3.6) | 2 (1.0) |
|  |  |  |  |
| **Duration of the chronic condition – mean (SD), years** |  | 10.2 (9.1) |  |
| **Duration of the chronic condition – median (IQR), years** |  | 7.0 [3.75 – 15.0] |  |
|  |  |  |  |
| **Long-term treatment – n (%)** |  |  |  |
| On a daily basis |  | 25 (89.3) |  |
| On a monthly basis |  | 3 (10.7) |  |
| No long-term treatment |  | 0 (0) |  |
| Does not wish to answer |  | 0 (0) |  |
|  |  |  |  |
| **Skipped the long-term treatment during the past month – n (%)** |  | **[N total =28]** |  |
| Never |  | 7 (25.0) |  |
| Less than once a week |  | 12 (42.8) |  |
| Several times a week |  | 7 (25.0) |  |
| Almost every day |  | 1 (3.6) |  |
| Never started the prescribed treatment |  | 0 (0) |  |
| Does not wish to answer |  | 1 (3.6) |  |
|  |  |  |  |
| **You feel free to discuss your treatment with your doctor, including your unwillingness to take it, potential side effects, etc – n (%)** |  |  |  |
| Yes, most of the time I can discuss these topics with my doctor. | 123 (50.0) | 17 (60.7) | 106 (48.6) |
| No, most of the time, I don't dare to talk about it. | 19 (7.7) | 3 (10.7) | 16 (7.3) |
| I don't feel concerned by this question | 101 (41.1) | 8 (28.6) | 93 (42.7) |
| I do not wish to answer | 3 (1.2) | 0 (0) | 3 (1.4) |
|  |  |  |  |
| **Number of visits to a doctor in the past year – n (%)** |  |  |  |
| > 10 times | 7 (2.8) | 2 (7.1) | 5 (2.3) |
| 5–10 times | 19 (7.7) | 7 (25.0) | 12 (5.5) |
| < 5 times | 143 (58.2) | 13 (46.4) | 130 (59.6) |
| Has not seen a doctor this year | 74 (30.1) | 6 (21.4) | 68 (31.2) |
| Does not wish to answer | 3 (1.2) | 0 (0) | 3 (1.4) |
|  |  |  |  |
| **Assessment of patient medication adherence – n (%)** |  |  |  |
| Rarely | 21 (8.5) | 0 (0) | 21 (9.6) |
| Depending on the patient and the medical situation | 113 (46.0) | 12 (42.9) | 101 (46.3) |
| Systematically for each patient with a long-term treatment and at each consultation | 112 (45.5) | 16 (57.1) | 96 (44.0) |
|  |  |  |  |
| **You would agree to use this device for yourself – n (%)** |  |  |  |
| Yes | 39 (15.8) | 7 (25.0) | 18 (8.3) |
| No | 166 (67.5) | 18 (64.3) | 148 (67.9) |
| I do not wish to answer | 11 (4.5) | 1 (3.6) | 10 (4.6) |
| Missing data | 30 (12.2) | 2 (7.1) | 28 (12.8) |

** For clarity, we report only the results for the 5 most prevalent chronic conditions. The results for all chronic conditions are detailed in Supplemental Table*
