# Supplementary material for: Acceptability of and Willingness to Take Digital Pills by Patients, the Public, and Health Care Professionals: Qualitative Content Analysis of a Large Online Survey
Source: J Med Internet Res. 2022 Feb 18;24(2):e25597. doi: 10.2196/25597 (PMC8900921; doi:10.2196/25597)
Supplement: Multimedia Appendix 8 [file jmir_v24i2e25597_app8.docx]

# Multimedia Appendix 8: Data saturation


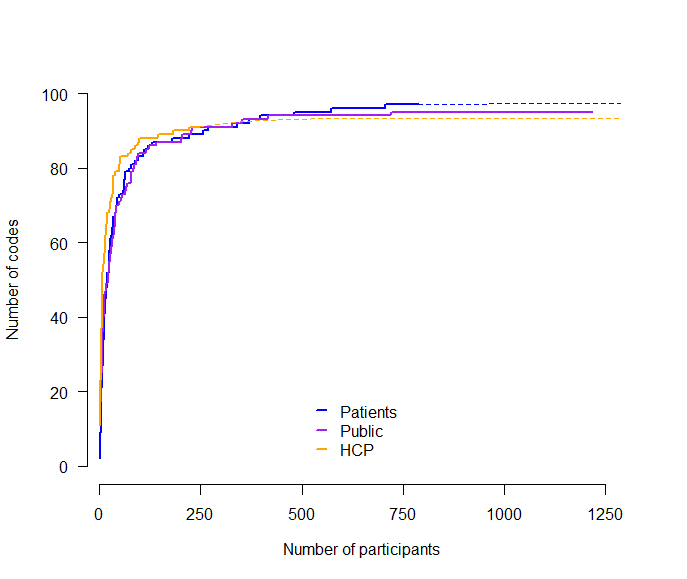


**Figure:** **Data saturation in patients (N=767), the public (N=1238) and HCPs (N=246).**

*Data saturation was reached up to 100% for public, 99.4% for patients, and 97.7% for HCPs.*
