# Supplementary material for: Acceptability of and Willingness to Take Digital Pills by Patients, the Public, and Health Care Professionals: Qualitative Content Analysis of a Large Online Survey
Source: J Med Internet Res. 2022 Feb 18;24(2):e25597. doi: 10.2196/25597 (PMC8900921; doi:10.2196/25597)
Supplement: Multimedia Appendix 9 [file jmir_v24i2e25597_app9.docx]

# Multimedia Appendix 9: Acceptability of digital pills

**Qualitative content analysis of the responses to the 5 open-ended questions about acceptability**

| **Themes and codes** | **Quotes** | **Patients**  **N=767** | **Public**  **N=1238** | **Total N=2005** | **HCPs**  **N=246** |
| --- | --- | --- | --- | --- | --- |
| **Perceived clinical effectiveness of digital pills - 29 codes** | | | | | |
| **Benefits of digital pills** |  |  |  |  |  |
| Prevent forgetfulness | “This helps to avoid forgetfulness. But is it the doctor's role keep the prescribed medication intake under surveillance? (patient, woman, 61 years old)  “It's a way to remind people when it's time to take their medication.” (patient, woman, 56 years old)  “If she gets an immediate reminder every time she forgets to take her medication, she will definitely take it more regularly.” (patient, man, 53 years old) | 98 (12.8) | 160 (12.9) | 258 (12.9) | 52 (21.1) |
| Prevent overdose | “She wouldn't forget it, and most importantly, she wouldn't make a mistake in taking them.” (patient, woman, 56 years old)  “If the patient is taking too much medication or not enough, they can be warned, so it's a good thing” (patient, woman, 47 years old)  “Useful if the patient cannot remember exactly how much he or she has taken, to keep the risk of error under surveillance.”(psychiatry, woman, 27 years old) | 16 (2.1) | 30 (2.4) | 46 (2.3) | 7 (2.8) |
| Prevent misuse | “The person will feel kept under surveillance and will therefore avoid overdosing on their treatment knowing that the doctor could find out very quickly.” (public, man, 28 years old)  “The patient knows that his doctor is taking his treatment and can't ask him for extra prescriptions to resell his medication.” (public, man, 18 years old)  “Good for better management of medicines because there is too much abuse in the reimbursement of medicines for greater transparency.” (patient, woman, 67 years old) | 2 (0.3) | 7 (0.6) | 9 (0.5) | 0 |
| Encourage treatment continuity | “You can't turn off the electronic. You could say it's a blessed step forward in the world of medicine. *A priori*, I have nothing against this concept. It can improve the regular and complete taking of the treatment. By taking this medication, one might feel a little more kept under surveillance and unable to cheat on taking the treatment. It would prevent me from stopping the treatment as soon as I feel better.”(public, man, 61 years old)  “I'm the first one, I often tend to stop my treatment as soon as I get better, I think many people do the same.” (public, man, 58 years old)  “Useful in pseudo-resistance to antidepressant treatment (among other things) because patients sometimes complain about the ineffectiveness of a product while it remains in the box without being taken. Or also for antidepressants in remission phase. Patients will often say, ‘Well, since I was feeling better, I stopped taking it’.” (psychiatry, woman, 27 years old) | 7 (0.9) | 7 (0.6) | 14 (0.7) | 1 (0.4) |
| Useful for assessing patient adherence | “This can be useful in cases where the doctor has doubts about whether the patient is complying with the treatment voluntarily or not (memory problems for example).” (patient, woman, 59 years old)  “The only good thing about this device is that the doctor is sure we are taking our treatment.” (public, woman, 35 years old)  “Gives information on the level of compliance in the event of ineffectiveness so that we do not rush into a dosage increase when we are looking at a lack of compliance. It also gives information on the ability to comply with the treatment alone, or the need to use IDE *(registered nurse)* pathways (elderly population, neuro-cognitive, mental, chronic illness...)” (psychiatry, man, 25 years old) | 20 (3) | 6 (0.5) | 26 (1.3) | 29 (11.8) |
| Useful for assessing drug efficacy | “The data received by the transmitter will be used to verify the effectiveness of the treatment.” (public, woman, 34 years old)  “I find it interesting to understand what happens if a treatment doesn't work.” (public, woman, 38 years old)  “I think it's a good idea for monitoring patients who have chronic illnesses to keep under surveillance. The data received by the transmitter will be used to verify the effectiveness of the treatment. I'll take it if it allows me to monitor my health and improve treatment for a chronic illness.” (public, woman, 34 years old) | 27 (3.5) | 49 (4) | 77 (3.8) | 1 (0.4) |
| Useful for assessing safety | “It would be useful to know about drug intolerances.” (public, woman, 80 years old)  “This type of device must be able to regulate the taking of medication in real time. There are often undesirable effects when taking certain medications being able to measure the effects can also perhaps reduce the dose and thus reduce the undesirable effects.” (patient, man, 59 years old)  “It's very ingenious for studying the response to medication right away. I am willing to try it for the speed of diagnosis through daily monitoring of the medication's progress. There will be no more taking a medication that sometimes destroys another place for nothing.” (public, woman, 42 years old) | 4 (0.5) | 7 (0.6) | 11 (0.6) | 6 (2.4) |
| Enhance treatment personalization | “I don't think the fact of connecting medications is useful unless the dosage of the medication can be changed according to connected analyses.” (public, woman, 45 years old)  “I will be volunteering to try it, I think it's a step forward... I hope it will lead to improvements for better optimisation of the treatment of illnesses.” (public, man, 46 years old)  “The person will be able to know exactly how her body responds to the treatment and whether her body still needs it. (public, man, 44 years old)  “Because each person is different and so is the way their body perceives and ‘uses’ the drug.” (public, woman, 29 years old)  “It could be useful, for example, for a Parkinson's patient with cognitive problems, poorly adjusted, to understand missing doses. This would help in choosing between switching to aids or switching to a non-oral device (pump). Or for example for an epileptic who is difficult to balance before escalating the therapy” (neurology, woman, 29 years old) | 6 (0.8) | 7 (0.6) | 72 (3.6) | 14 (5.7) |
| Reduce face-to-face consultations | “I find this concept very modern and sometimes useful if patients cannot travel to see a doctor, then a remote consultation is possible.” (public, woman, 46 years old)  “However, this device could be very useful for patients with problems who forget to take their prescription. This would avoid in some cases the need for a nurse to come to the home just for taking the medicines.” (public, woman, 24 years old)  “This allows regular monitoring without going to the doctor's surgery, thanks to quick access to data.” (public, woman, 34 years old)  “It seems more logical to me to look inside the body to see what is wrong and not having to go myself every time could be a real advantage.” (patient, man, 42 years old) | 28 (3.7) | 48 (3.9) | 76 (3.8) | 4 (1.6) |
| Reduce additional testing | “If the data collected are more accurate and avoid further tests I would be positive enough to try.” (patient, man, 58 years old)  “It’s not bad. One avoids unnecessary consultations. One would avoid blood tests to monitor the dosage of medication.” (public, man, 56 years old)  “This helps to check that the patient is taking the medication, but does this capsule have other functionalities? If so, it would avoid some intrusive examinations and costly hospitalisation. If the data collected are more accurate and avoid further tests I would be positive enough to try it. Telemedicine avoids a trip to the doctor's surgery, unless necessary as a result of the data transmitted.” (patient, man, 58 years old) | 6 (0.8) | 8 (0.6) | 14 (0.7) | 2 (0.8) |
| Improve overall follow up | “It allows us to monitor the effectiveness, which is already good! and also that the doctor cares a little bit about his patient through closer monitoring.” (patient, man, 57 years old)  “There will be a much more effective monitoring of treatment by the doctor, as opposed to sporadic visits.” (public, man, 61 years old)  “The family doctor or specialist may monitor the illness on a day-to-day basis, whether good or bad, and may modify treatment or dosage if there are any problems, undesirable side effects or worsening of the patient's health. In addition, this device could also alert to new complications or illnesses that were not previously detected and that could be managed more quickly.” (public, man, 74 years old)  “May allow the doctor to ensure that certain ‘at-risk’ patients, such as the elderly, take their medication and provide appropriate monitoring.” (public, woman, 27 years old) | 105 (13.7) | 170 (13.7) | 275 (13.7) | 9 (3.7) |
| **Potential added benefits** |  |  |  |  |  |
| Could refine the diagnosis | “Interesting to understand the evolution of an illness depending on whether or not the medication is taken and when it is taken (e.g. blood glucose...)” (patient, woman, 24 years old)  “Interesting because it allows you to see the efficiency and if the problem has been localised.” (public, woman, 53 years old)  “This would allow for a more thorough medical examination and a better assessment of needs.” (patient, woman, 24 years old)  “This could detect abnormalities that current medical devices are not yet able to detect and increase the accuracy of diagnosis for the physician.” (patient, woman, 44 years old) | 16 (2.1) | 24 (1.9) | 40 (2) | 0 |
| Could screen other health problems | “Ideally, there should be a camera.” (public, man, 65 years old)  “Interesting if you can combine the different pills to be taken in a single one. One could consider an equivalent technique that exists in the context of an examination such as in gastroenterology to find other problems.” (patient, man, 64 years old)  “Constant checking of people's health. It's a crazy thing if it's just to check the patient's treatment. To detect an illness, it would have shocked me less. It opens up other perspectives that I think are more useful.” (public, woman, 42 years old) | 18 (2.3) | 31 (2.5) | 48 (2.4) | 6 (2.4) |
| Could be connected to a critical care facility | “This could also serve as a warning system for the doctor (fall, fainting, discomfort, etc.) he could be in direct contact with the patient in case of a problem.” (patient, man, 62 years old)  “For serious illnesses, the risk of cardiac arrest, that may be of interest. But in this case with a connection to the accident and emergency department or the fire brigade.” (public, man, 38 years old)  “This device would be suitable for certain high-risk pathologies such as heart and respiratory problems. The doctor would have access to alerts in case of risks for the patient. I would take it for my own safety if it turned out that I had a serious pathology.” (patient, man, 72 years old)  “This could also serve like a warning system for the doctor (fall, fainting, discomfort, etc.) he could be in direct contact with the patient.” (patient, man, 62 years old) | 4 (0.5) | 5 (0.4) | 9 (0.5) | 0 |
| **Mechanisms for improved adherence** |  |  |  |  |  |
| Smartphone schedule reminder | “There must be alerts on the phone to know exactly when to take them.” (public, woman, 31 years old)  “I imagine there's some kind of vibrating device or something that reminds you to take the medication.” (public, woman, 76 years old)  “The patch reminds you to take your medication, to swallow a device as well. The innovative aspect makes it a reminder that one does not forget.” (nurse, woman, 38 years old)  “Rapid feedback encourages compliant behaviour. By simply measuring a behaviour, it can be improved.” (psychiatry, woman, 41 years old) | 96 (12.5) | 122 (9.9) | 218 (10.9) | 22 (8.9) |
| Prompt a phone call reminder from the doctor | “The doctor could also warn the patient if he finds that the protocol is not being followed.” (public, woman, 38 years old)  “If she forgets, she can receive an alert from the doctor, or even automated by the smartphone app.” (public, man, 54 years old)  “The doctor could send a reminder, via a text message system for example, to tell the patient that he has not taken his medication and that it is important to do so.” (patient, woman, 41 years old) | 4 (0.5) | 9 (0.7) | 115 (5.7) | 5 (2) |
| Fear of being argued with by the doctor | “I suppose the fear of being ‘reprimanded’ by the doctor might make encourage some people.” (patient, man, 48 years old)  “She might ‘be afraid’ of being scolded a little by her doctor who might see that she hasn't been taking the treatment he has prescribed.” (patient, woman, 41 years old)  “She would feel 'monitored' and 'spied on' by her doctor. This system works on ‘Fear of the policeman’ as the expression goes.” (public, woman, 27 years old)  “The patient will take his treatment for fear of being unmasked, reprimanded, because it will be difficult for him to defraud without being seen. He will also have the financial fear of a reimbursement in case of poor compliance.” (cardiologist, man, 28 years old) | 45 (5.9) | 60 (4.8) | 105 (5.2) | 44 (17.9) |
| Anticipated guilt/shame | “She would feel as if she wasn't being honest with her doctor.” (public, man, 54 years old)  “If she feels that she' s being tracked, it will make her feel responsible but also guilty.” (public, woman, 28 years old)  “She will feel guilty if she doesn't take it.” (patient, man, 64 years old)  “She would be ashamed not to take them and would no longer be able to lie to her doctor!” (patient, woman, 83 years old)  “The patient wouldn't want to make a fool of himself in front of the doctor and would take his medication.” (public, woman, 62 years old)  “The patient will be complying out of shame that the objective physician actually forgets and out of fear of being reprimanded.” (psychiatry, woman, 26 years old) | 5 (0.7) | 3 (0.2) | 8 (0.4) | 10 (4.1) |
| Social desirability | “She will be proud to know when her doctor sees that she hasn't forgotten him (but it's a bit childish).” (public, woman, 60 years old)  “I agree to take it to avoid forgetting and to show that I'm taking care of myself properly.” (public, man, 27 years old)  “She would be afraid of being mistaken for an irresponsible negligent person.” (public, man, 71 years old)  “I am willing to take this that way my doctor can trust me even more.” (public, woman, 44 years old)  “I will take it so the doctor can see how disciplined I am.” (patient, woman, 52 years old)  “The Hawthorne effect, but also the fact that you discussed it with your doctor, that you took the time to answer the patient's questions.” (general practitioner, man, 31 years old)  “Treatments will be taken because the patient may feel ‘controlled’ and kept under surveillance in terms of their medication intake and may want to be ‘good’?” (general practitioner, woman, 25 years old) | 9 (1.2) | 4 (0.3) | 13 (0.7) | 19 (7.7) |
| Pressure of surveillance | “The fact of being observed by the doctor even from a distance makes the patient more scrupulous about his treatment. He can no longer lie.” (public, man, 61 years old)  “Knowing that they are being ‘monitored’ may unconsciously lead the patient to better follow his treatment.” (public, man, 37 years old)  “Big Brother is watching you. If the patient knows that the doctor can tell whether or not the medicine is being taken, he will take it.” (public, man, 46 years old)  “The fear of being kept under surveillance; but, except for one very very particular reason (Alzheimer's, hyper ‘sensitive’ medication), it is being treated like a child, I find it, and even degrading.” (patient, woman, 71 years old)  “The person would take their treatment because they would know that the doctor has access to the actual taking. It is a social pressure: the doctor has particular expectations that the patient will want to respect.” (patient, woman, 19 years old)  “Because of the pressure of kept under surveillance, of being picked on like a child who hasn't done her homework, she will take her medication.” (public, woman, 27 years old)  “I don't see the point:  - if there's a risk of forgetting, there are many simpler solutions (note in diary, reminder on mobile phone)  - if I do not take the treatment because I am not convinced of its usefulness or because I am afraid of possible side effects, I will feel obliged to take it... And that, in my opinion, poses an ethical problem because it's a form of pressure that harms the patient's autonomy.” (general practitioner, woman, 34 years old) | 77 (10) | 141 (11.4) | 218 (10.9) | 73 (29.7) |
| Fear of penalty | “It feels like a search for an error so that the insurance company will no longer take over the treatment.” (public, man, 58 years old)  “This is already used for sleep apnoea devices. But it doesn't serve the purpose of better monitoring the patient's treatment. The data are collected every 6 months by a technician and not a doctor. There is no recommendation, it serves only for the company that provides the equipment to be able to justify the use of the equipment to the health authorities. My prescribing doctor cannot physically go and see the data (number of patients too large). He would have to pilot a big data system...” (patient, man, 56 years old)  “If there's no adherence to the treatment, the doctor will know right away. Won't social security still take advantage of this to ‘police’ patients more? For example, if a medication is forgotten, it will no longer be reimbursed.” (patient, man, 52 years old)  “The person will take their treatment for fear of being hospitalised if they don't take the medication on their own.” (public, woman, 38 years old)  “Fear of negative repercussions in the event of poor compliance, in particular a cessation of treatment by the doctor (for expensive treatments) or a stop placed on reimbursement.” (haematology, woman, 31 years old) | 14 (1.9) | 21(1.8) | 35 (1.8) | 26 (10.5) |
| **Potential harms or futility** |  |  |  |  |  |
| Sensor safety | “I don't like the idea at all. Personally, having a transmitter in my body doesn't reassure me at all. I wouldn't trust that kind of system.” (public, woman, 18 years old)  “It is not clear whether the technology ingested can negatively affect the patient's health.” (public, man, 32 years old)  “I'm afraid my body won't reject the transmitter.” (public, woman, 58 years old)  “I'm afraid of not being able to tolerate the device in the digestive tract... allergy or reaction.” (patient, man, 69 years old) | 190 (24.8) | 415 (33.5) | 605(30.2) | 60 (24.4) |
| Radiation safety | “We are already living in a thick fog of all kinds of waves that are imposed on us and now, on top of that, they want to make us swallow them!” (patient, woman, 61 years old)  “If this medication is not dangerous, but that is not the case with this technology, the technology must cure not make you sick. The body is subjected to electromagnetic radiation.” (patient, man, 46 years old)  “There is zero or no recoil on the effects of Bluetooth waves on the body.” (patient, man, 51 years old)  “I don't want electronics in my body. Have any serious studies been done on the waves emitted?” (patient, man, 58 years old) | 102 (13.3) | 212 (17.1) | 314 (15.6) | 32 (13) |
| Could worsen mental health disorders | “In psychiatry, the sensor can fuel delusions of past, present or future persecution.” (psychiatry, man, 28 years old)  “It will be a factor of psychotic decompensation, increase persecution, feeling manipulated” (psychiatry, woman, 46 years old)  “Schizophrenic patients often have delusions about machines in the body, physical or mental invasions, hidden surveillance devices... the device described could freak them out.” (psychiatry, woman, 27 years old)  “Risk of feeling persecuted for paranoid or sensitive patient.” (nurse, man, 35 years old) | 0 | 0 | 0 | 21 (8.5) |
| Interaction with other devices | “How does it work? Is there a risk for those who have a pacemaker, for example?” (public, man, 32 years old)  “Are the medication's emissions harmful to the patient who, in fact, doesn't need anything more to make their health worse. Could it disrupt his pacemaker, for example?” (public, woman, 60 years old) | 2 (0.3) | 13 (1.1) | 15 (0.8) | 3 (1.2) |
| Poor acceptability | “I don't know if the person will take her treatment better. I think she will have less chance to complain about her health if the doctor sees that she is not taking her treatment properly. I am thinking in particular of my grandmother who complains about her blood pressure but who doesn't take the medication every day because she saw on the leaflet that it could cause cystitis as a side effect. That doesn't stop her from complaining to the doctor about her blood pressure...” (patient, woman, 36 years old)  “I doubt that patients who take liberties with their treatment will stick with the device.” (patient, man, 66 years old)  “There's no reason for a person to take it more with than without a transmitter.” (public, man, 42 years old)  “That's the problem: if a person doesn't follow a prescription, why would they want it to be known that they're doing whatever.” (patient, woman, 59 years old)  “As for a person in denial of his illness and the care that goes with it, it's not sure he'll accept it any better with this policing.” (patient, woman, 66 years old)  “For the patient, it will be necessary to show great pedagogy so that the device does not pass for a policeman who does not give his name ... the patient must also find it advantageous, otherwise non-compliance will be reported in a different way with the risk of losing the therapeutic link with the practitioner.” (general practitioner, man, 33 years old)  “In psychiatry, this process can be complicated for patients who are very often suspicious and persecuted, and all the more so if ‘something enters their body’ to keep them under surveillance... For the elderly, connected tools are not news except for the next generation.” (nurse, woman, 51 years old)  “I don't really see the point of such a complex system, when most of the information can be gathered through interrogation. The patients who are going to accept this device will probably be compliant patients and not the most problematic ones.  Compliance is also a matter of education and not ‘policing’. I don't see how being constantly kept under surveillance is going to get the patient to take their medication, other than by telling them they're going to get shouted at by their doctor, which is not our role.” (dermatology, woman, 26 years old) | 24 (3.1) | 22 (1.8) | 46 (2.3) | 42 (17) |
| It won’t improve adherence | “If I am ill, I am free and responsible to treat myself or not to treat myself commercially: we have found an enormous new ability for the naive to commercialise.” (public, woman, 58 years old)  “The therapeutic interest seems to me to be low compared to the rather complex system to set up and the cost.” (patient, woman, 58 years old)  “If I don't feel like taking the last two days of antibiotics, for example, this is not going to force me to do it.” (patient, woman, 58 years old)  “It's stupid to stick a patch on yourself: if you forget treatment, you forget patch.” (public, woman, 62 years old)  “Not sure how effective such a system would be. If a patient doesn't take his treatment, because of lack of confidence or because he forgot, will the doctor will call his patient to remind him? He has something else to do, I think, given the number of hours he's working...” (public, man, 61 years old)  “In the short term, like any new product, it may have a positive effect on the person, with controlled monitoring, but, over time, I think that, as with any prescription, the person will not necessarily connect to their mobile phone and will delete the application linked to this system.” (public, woman, 31 years old)  “I don't think this will have any impact on compliance, except for the one due to a one-time lapse in compliance. For non-compliance due to reluctance, mistrust, undesirable effects, etc., there will be no improvement because the system does not allow the reason for non-compliance to be highlighted.” (pharmacist, man, 31 years old)  “I'm not sure policing really serves to improve compliance... The manufacturer says that because he wants to sell it... But I don't think I have seen a scientific study (was it commissioned by the lab) that actually proves that it improves the patient's life.” (psychiatrist, man, 35 years old)  “Clinical uselessness of such a device (do we really need keep our patients under surveillance in this way?) Should the medicine replace the common sense of patients who are offered treatment? The doctor's role is upstream to prevent non-compliance, not downstream to police the patients). What are the real interests of the manufacturers offering the technology? Generally, philanthropy is not their strong point (creation of a giant database to be sold to the highest bidders? Targeted products based on data collection?) There is also a risk of medical over-consumption by offering the patient the possibility of direct access to crude health parameters, without explanation.” (general surgery, man, 32 years old) | 117 (15.3) | 162 (13.1) | 279 (13.9) | 26 (10.6) |
| Useless gadget | “One more device to clutter up smartphones.” (public, man, 71 years old)  “I don't need this gadget to take my treatment seriously.” (public, man, 71 years old)  “It seems to ask more questions and problems than it seems to solve.” (public, man, 19 years old)  “A foreign body in a medication just to prove to your doctor that you're taking the medication correctly: I don't see how that's going to help us patients. I wouldn't take that. I don't need a transmitter in my body to prove to my doctor that I've taken my medication properly” (patient, woman, 25 years old)  “It's the bond of trust that needs to be worked on. Not raving about New Age gadgets.” (nurse, man, 59 years old) | 97 (12.6) | 158 (12.8) | 255 (12.7) | 64 (26) |
| Better alternatives for adherence exist | “I think it could be useful, but in very few cases. This kind of device, very expensive in terms of effort (and probably in terms of cost) will be useful for patients e.g. with Alzheimer's who can frequently forget about medications. In serious cases it is necessary to have a nurse to control the medication, but in the beginning, perhaps this kind of expense can be avoided with a remote control with this kind of device.” 1 (patient, man, 73 years old)  “I can do it very well with an alarm on my phone (e.g. for my pill)... Why, yet again assisting people?” (public, woman, 42 years old)  “If people have Alzheimer's disease, for example, they have to go to an old people’s home or at least have some personal assistance. In addition, there is a very simple solution for people who forget their medication too often: the pill box for the week.” (patient, woman, 64 years old)  “A person who takes their medication on a regular basis will continue to take it regularly, whereas a person who is a little distracted in taking their medication will not change their habits. It would be better to have a telephone programmed to remind the patient that it's time to take their medication.” (patient, woman, 77 years old)  “I don't want it to come to this. An alarm or a memo will be more than enough!” (patient, man, 34 years old)  “It's multi-professional therapeutic education that needs to be done!” (public, man, 28 years old)  “This approach is the opposite of Therapeutic Education, which aims to educate the patient, to empower them. Here, this is policing pure and simple... No pedagogical aspects. Might as well put an alert on the smartphone to remind the patient to take it, quite simply ... " (general practitioner, man, 31 years old) | 20 (2.6) | 16 (1.3) | 36 (1.8) | 36 (14.6) |
|  |  |  |  |  |  |
| **Perceived burden of digital pills– 6 codes** | | | | | |
| **For the patient** |  |  |  |  |  |
| Complexity | “It seems to me to be quite heavy in the sense of the many objects involved in the device.” 106734852 (patient, man, 46 years old)  “On reflection, I think it may be useful for a certain public: the elderly, cognitively impaired or mentally ill who regularly forget their treatment. It would allow doctors to better understand the patient's reactions. But I find it complicated to understand, for the public, for whom it could be useful (Bluetooth, patch to stick, smartphone...) " (patient, woman, 28 years old)  “Too many constraints, too much risk-taking, to end up just saying to my doctor, "That's it, doctor, I've taken my medicine".” (public, woman, 27 years old)  “There are too many negative points: the obligation of a patch, the obligation to have a dedicated app, the obligation of the agreement for access to data, it's very intrusive.” (public, man, 50 years old)  “Total lack of usefulness!!!! The complexity of implementation (sticking the patch...), that Bluetooth works, that the patient uses the smartphone...” (general practitioner, man, 36 years old) | 32 (4.2) | 46 (3.7) | 78 (3.9) | 13 (5.3) |
| Cumbersome | “The patch can be uncomfortable for people who wear it. It is important to know how long the patch lasts and whether it is water-resistant because people who forget their medication may also forget to put the patch on if it needs to be changed regularly.” (public, woman, 24 years old)  “The patch may peel off with sweat, heat and water in the shower.” (public, woman, 42 years old)  “The fact of ingesting a transmitter seems strange to me. Also, I don't feel like having a patch stuck to my skin all the time.” (patient, woman, 41 years old)  “Ingesting a medication on a daily basis is already restrictive. Moreover, having a patch and the app on your phone is more constraining and there is nothing to say that the doctor will take the time to read and contact the patient in the end.” (patient, woman, 55 years old)  “Patches all over the body, especially in the summer, are unsightly... and too bulky.” (patient, man, 59 years old) | 34 (4.4) | 41 (3.3) | 76 (3.8) | 14 (5.7) |
| Permanent connection | “It is unbearable to always be on a super-connected cell phone...” (public, woman, 73 years old)  “You still have to have your cell phone, and that's still a constraint.” (public, woman, 48 years old) | 35 (4.5) | 36 (3) | 71 (3.5) | 12 (4.9) |
| Too difficult for some people | “The idea of ingesting a ‘technological’ product can be upsetting to many people, especially older people who may not understand the usefulness of the device.” (public, woman, 24 years old)  “It is necessary to have a smartphone and I am not sure that the device would be suitable for an older public (who are more likely to need it).” (public, man, 24 years old)  “There will be better monitoring if the sick person is a mobile phone enthusiast: true for the young, less obvious for the elderly.” (patient, man, 71 years old)  “I find it hard to imagine someone who needs monitoring of their medication intake (dependent person, elderly, etc...) having such a patch device / smartphone.”) (patient, man, 36 years old)  “Too connected, too tedious, and if the patient has cognitive problems, there is a risk they may remove the patch.” (general practitioner, woman, 29 years old) | 12 (1.6) | 14 (1.1) | 26 (1.3) | 15 (6.1) |
| **For the doctor** |  |  |  |  |  |
| Increases workload | “It is useless and the doctor has other things to do than making sure the patient has taken his medication.” (patient, woman, 38 years old)  “The doctor also becomes a slave to his patient: doesn't he currently have enough work to do without this extra burden being imposed on him? What is the purpose of this? Will the doctor have to notify his patient if he has not taken the prescribed medication?” (patient, woman, 61 years old)  “Finally, have mercy on the doctors! They have enough work already. Do they have to be at our service 24 hours a day, be informed in the middle of their family meal or... during their conjugal duty that little Mrs Martin has taken her pill? And what will the doctor's power be if the medicine isn't taken? Will he phone little Mrs Martin to remind her that she has a pill to take?” (patient, woman, 61 years old)  “I would accept the approach and the system if the monitoring followed because doctors and specialists are currently quite overbooked and may have little time to follow the day-to-day evolution of their patients' illnesses.” (patient, man, 74 years old)  “It is not knowing everything to use a technological method such as this ... And what is the precise benefit of having the information in question? (be it on the patient's side, on the doctor's side and on the ‘medication’s’ side). It seems an unnecessary cost to generate data that is not exploited or not exploitable. On the practitioner's side it adds yet another interface/another additional device (it will probably not be directly integrated into the business software). What impact can be expected on the practice?” (general practitioner, man, 33 years old)  “What about the doctor who has the results in real time day and night? And if the doctor doesn't react, what about his responsibility? "(anatomical pathology, woman, 56 years old)  “Either the patient sees a benefit in taking a treatment and will eventually take it, or he doesn't and will stop anyway. What do I do when I see on my phone that he has stopped? Do I send the police to him?” (general practitioner, man, 43 years old) | 62 (8.1) | 65 (5.3) | 128 (6.4) | 37 (13) |
| Additional work remuneration | “She could not exceed the prescribed doses, or forget them, without being called to order by the doctor, who would probably bill her for the service rendered.” (patient, man, 55 years old)  From a theoretical point of view, the idea may sound good, but will the doctor take the time to monitor this information ‘in real time’ in addition to his consultations? If several patients are being monitored at the same time, the doctor may take some time during the day to monitor the data, but not all patients take their treatment at the same time... Some mornings, noon and evenings, others once a week. How will the doctor invoice for this time?” (public, man, 62 years old) | 1 (0.1) | 1 (0.1) | 2 (0.1) | 0 |
|  |  |  |  |  |  |
| **Perceived ethicality of digital pills – 25 codes** | | | | | |
| **Values attached to technologic device** |  |  |  |  |  |
| Progress | “I will try it because I have the culture of new technologies” (public, man, 34 years old)  “I am for because I believe in modernity and artificial intelligence” (public, man, 43)  “I love technology and this medication is a big breakthrough” (public, man, 48 years old)  “I am very new technologies in all fields” (public, man, 54 years old)  “It's an advance to have a transmitter in the body to send information.” (public, man, 55 years old)  “You have to live with the times, therefore with progress.” (public, man, 65 years old)  “Good innovation, unfortunately society may not be open-minded enough yet. That being said, I prefer to wait until it really works well.” (patient, woman, 21 years old)  “Only by accepting certain methods can one advance in medicine.” (nurse, woman, 46 years old) | 59 (7.7) | 131 (10.6) | 190(9.5) | 29 (11.8) |
| Credibility | “It's interesting, it's as close to reality as possible and probably the truth.” (public, man, 31 years old)  “One positive point is that it is impossible to cheat on the intake of the treatment.” (public, man, 61 years old)  “There will no longer be any doubt about taking or forgetting to take medication, and the monitoring by the doctor that imposes honesty on the patient.” 7 (public, man, 39 years old)  “Doctors would be well informed, better than directly by their patient” (patient, woman, 71 years old)  “There would be a proof and verification of the correct intake of the treatment” (public, man, 34 years old)  “This helps to avoid the medical errors that often occur when a patient lies about taking their medication.” (public, man, 29 years old)  “Better monitoring of compliance by the doctor, the patient cannot lie about taking it.” (general practitioner, woman, 29 years old) | 12 (1.6) | 30 (2.4) | 42 (2.1) | 9 (3.7) |
| Entertainement | “I'm willing to try and see the results, but if it's not conclusive I won't continue.” (patient, man, 58 years old)  “If it's well developed and there are no contraindications, I will be curious about testing it” (patient, woman, 64 years old)  “I like to test innovations of all kinds” (public, woman, 60 years old)  “Agree to take it, out of curiosity about testing a new technology.” (public, man, 39 years old)  “Innovative and fun device. The fun side of this could reinforce patient compliance” (man, general practitioner, 28 years old)  “Playfulness and possible gamification to motivate compliance” (psychiatrist, man, 37 years old) | 49 (6.4) | 56 (4.5) | 105 (5.2) | 10 (4.1) |
| Mistrust | “I don’t trust an electronic device in my body” (public, woman, 56 years old)  “I think we are already connected enough that we are adding foreign bodies into our bodies and I think it is useless and dangerous. I think it is a concept that should not be born.” public, man, 23 years old)  “I refuse all connected devices. Question of possible hacking and intrusion into my private life.” (public, woman, 63 years old) | 41 (5.3) | 75 (6.1) | 116 (5.8) | 8 (3.3) |
| Reduce self-efficiency | “Dependent on several objects: telephone, object inside the body. No more reflection or thinking (soon to go to the toilet we will also depend on a connected object!).” (public, woman, 61 years old)  “I don’t want to rely on a machine. I don’t want to take responsibility away from myself” (public, man, 76 years old)  “This will create a dependency on connected systems, with all the related problems of energy cost, financial cost, misuse of data.” (general practitioner, man, 25 years old) | 2 (0.3) | 6 (0.5) | 8 (0.4) | 5 (2) |
| Inequality in access | “Necessary to have a smartphone so this is not suitable for disadvantaged populations.” (public, woman, 32 years old)  “You have to have a smartphone and be in an area with coverage and not in a white zone!” (public, woman, 62 years old)  “What about use when you have an old mobile phone” (patient, man, 64 years old)  “I know a number of people who are not capable of using a smartphone” (public, man, 71 years old)  “Everyone needs to have a laptop, which is a problem for people who are uncomfortable with technology” (public, woman, 55 years old)  “And what if you don’t have a smartphone? Will there be a free one issued by Social Security?” (patient, man, 61 years old)  “I don’t subscribe to this system at all and what’s more, as far as my mobile phone is concerned, I only have a subscription for €2 without internet access because my budget doesn’t allow me to take out an internet subscription and what’s more I’m not constantly tied to my phone.” (public, woman, 68 years old)  “I think its a forward-looking device for modern medicine. The only problem is that it might only concern young or not-so-old patients with smartphones, connected to the internet and that the elderly who are more often ill would be left by the wayside except for the use in EHPADs with centralised information collection. I don't see any negative points if everyone is taken into account.” (patient, man, 74 years old)  I am torn between enthusiasm for these new technologies and the consequences that this implies. Especially in terms of privacy and access to personal data, as well as the security of this data at all levels. But these are still problems that can be found in all areas using connected objects. I also wonder about who can be prescribed this type of device. Not everyone has a smartphone or knows how to use one. The problem of compliance, in my opinion, will concern a category of the population that is likely to have difficulties with this type of device. (general practitioner, man, 31 years old) | 45 (5.9) | 36 (2.9) | 81 (4) | 10 (4.1) |
| Pollution | “Once the device and its components are evacuated, they go ‘into nature’, creating a risk of water pollution” (public, woman, 43 years old)  “Technology is not good for the body or the environment, it's encouraging them for something rather futile” (patient, woman, 19 years old)  “I'd be afraid there would be something left in the body. Connected objects are not healthy for health and the planet.” (patient, woman, 68 years old)  “It is a waste of a planetary resource for a device that will end up in the sewer” (public, man, 40 years old)  “What is the impact of the patch on health? And the environmental impact (recycled? Eliminated? How? Associated pollution?) (anaesthetist, woman, 34 years old) | 23 (3.0) | 44 (3.5) | 67 (3.4) | 6 (2.4) |
| **Value attached to human rights** |  |  |  |  |  |
| Liberty | “I hate the idea of giving control over my health care to an external ‘entity’” (patient, woman, 66 years old)  “I don't like this kind of control at all, it hinders our last freedoms.” (patient, woman, 64 years old)  “I want to maintain my freedom of choice” (patient, woman, 64 years old)  “It is a loss of freedom, it is the manufacturer who is taking control. There must be no link between a manufacturer and a user. Up to now, a user has been free to make his own choices and this type of product takes away his free will. For my part, I will never take this type of product.” (patient, woman, 66 years old)  “This is an infringement of individual liberty and a question of ethics. The manufacturer is in it for financial profits and the patients have the right to choose. These are two parallel logics.” (nurse, man, 50 years old) | 38 (5.0) | 31 (2.5) | 69 (3.5) | 43 (17.5) |
| Intrusiveness | “This is spying from inside my body. I would be worried about the side effects too.” (patient, woman, 64 years old)  “There is far too much indiscretion and invasion of privacy” (public, woman, 55 years old)  “I find it intrusive, I don’t want my doctor to know if I took my medication and at what time” (public, man, 48 years old)  “It is too intrusive: taking medication is personal” (public, man, 52 years old)  “Intrusion of the medicine into the intimacy of the human being.” (public, man, 47 years old)  “It’s horrible! It feels as if it’s really touching on intimacy. Ethically, it is frightening. That we use applications is one thing because we can limit its use in, in time, but a pill that we ingest is really to futuristic and too invasive.” (public, woman, 33 years old)  “It is a categorical refusal, I still prefer to take a drug that has less effect but I will never consciously swallow a capsule that allows other people to follow me or my lifestyle, even if already in society we are monitored in many areas without knowing it, my doctor has the right to suggest it after it is up to the person to accept it or not” (patient, woman, 44 years old)  “Very intrusive for the patient, but ideal for the practitioner.  A coercive device, however, which can be likened to ‘policing’ and therefore potentially reserved for injections of transmissible or expensive therapies.” (cardiologist, man, 28 years old) | 66 (8.6) | 150 (12.1) | 216 (10.8) | 62 (25.2) |
| Dehumanization | “I'm having a little trouble accepting the fact that I'm connected to all the systems today. I think in a lot of cases we are not free anymore. We are under control. What are we going to be in a few years? Machines being kept under surveillance by other machines! And the freedom in all that!” 14220914 (public, woman, 62 years old)  “I want to retain my free will. This does not respect autonomy. There's not enough of the human in this treatment.” (patient, woman, 66 years old)  “We're already guinea pigs and we're becoming robots... “(patient, man, 64 years old)  “The person who takes it becomes a robot or a sheep.” (public, woman, 27 years old)  “If I took this I'd feel like a remotely controlled robot.” (public, woman, 51 years old)  “I refuse to take this to avoid being made robotic and irradiated by these waves. The ones I am already subjected to in spite of myself are already, ethically, beyond what I can bear.” (patient, woman, 61 years old)  “No, really, I don't want that. It makes me think of microchipped animals.” (public, woman, 71 years old)  “When you're sick, you're already dispossessed of your 'medical' life, especially in a hospital environment, so with that on top of it, no thanks.” (patient, woman, 53 years old)  “It sounds complicated, the man becomes a connected subject like any other, a benefit for sick people who are difficult to monitor.” (public, woman, 36 years old)  “No thanks, I won't take any connected medication, I don't want to be kept under surveillance that much. It contributes to the dehumanisation of our society.” (neurologist, woman, 29 years old) | 32 (4.2) | 46 (3.7) | 78 (3.9) | 41 (16.7) |
| Policing | “The illness and the taking of medication are stressful enough as it is, so there's no need to add a layer of surveillance on top of it.” (patient, woman, 33 years old)  “It would be one more step towards the traceability of the individual; I'm not sure I'd subscribe to it.” (public, man, 64 years old)  “It might be a way to make sure an older person is taking treatment, but it's policing, in that sense it sends back something very negative and unhealthy (loss of trust in a patient). What may be of interest to a patient is if they are given new data related to this device (monitored by a measurement if the transmitter is analysing things in the body at the same time).” (patient, man, 44 years old)  “No, I’m against it. We are still a country of free expression and not in a dictatorship as far as I know, that wants to check what is not done and done, one is already kept under surveillance on the cash one takes from ATMs, and that's all it would take to be kept under surveillance on the taking of medication.” (patient, woman, 47 years old)  “Negative point: “Big brother is watching you...” (public, man, 61 years old)  “It's a bad idea, unethical. The question really arises with some expensive treatments, and some doctors already think that they should stop prescribing them or reimbursing poor adherents. But how do we define good compliance? It is a dependant medication, in terms of effectiveness, and therefore complicated to manage. There will be a feeling for patients of being kept under too much surveillance, and a loss of confidence in doctors.”(haematologist, woman, 31 years old) | 112 (14.6) | 143 (11.6) | 255 (12.7) | 90 (36.6) |
| **Value attached to medicine** |  |  |  |  |  |
| Infantilization of the patient | “What's the point? To make sure we're grown up enough to take our medicine properly?” (patient, woman, 41 years old)  “The person will take his treatment ‘better’ through FEAR, because he will be in a worse than sectarian grip. They will be treated like a child and will be afraid of being ‘scolded’ by their doctor.” (patient, woman, 61 years old)  “I think a 'normal' person doesn't need to be treated like a child. (patient, woman, 75 years old)  “If she was taking her medication better with this device, I would think it was a shame and a bit childish.” (public, woman, 65 years old)  “the per son will take the treatment because she feels she under surveillance, so she feels compelled to do it. It can therefore be said that it is pushing people into dependency and/or babysitting on a large scale.” (public, woman, 26 years old)  “I wouldn't want my doctor to interfere with my private life. It’s being treated like a child.” (public, woman, 46 years old)  “It is removing responsibility from patients, treating them like children. The doctor becomes a controller.” (general practitioner, woman, 31 years old) | 20 (2.6) | 15 (1.2) | 47 (2.4) | 29 (11.8) |
| Disempowerment of patients | “I am fit to vote, read, sign by and care for myself.” (patient, man, 71 years old)  “I am independent, I take my treatment regularly, I don't need to be controlled.” (patient, woman, 71 years old)  “A little strange, though. An adult is responsible for his own treatment and is therefore free to monitor his treatment.” (public, man, 18 years old)  “I have to be responsible for my decisions. I don't need anyone, let alone a device that reduces MY freedom!” (public, man, 67 years old)  “Taking responsibility away from the patient. The patient becomes passive rather than active in his recovery. A device suitable for patients who are considered unfit to take responsibility for monitoring their own treatment.” (public, woman, 40 years old)  “Not at all agree: too much connection, not enough responsibility. We become robots” (patient, woman, 61 years old)  “I find it strange that it is the doctor who controls the taking of medication...isn't it the patient's responsibility?” (public, man, 55 years old)  “This will make our patients even less independent and responsible, which does not promote self-normativity in the illness. It also shows a lack of confidence.” (nurse, woman, 38 years old) | 44 (5.7) | 44 (3.6) | 88 (4.4) | 30 (12.2) |
| Inconsiderate to patient’s perspective | “Very bad idea, we're in a dictatorship. People are free to take care of themselves or not. Even sick people are free.” (patient, man, 75 years old)  “Again, the use of new technologies even for health we can also chip humans, so we can keep them under surveillance totally!!! Health is a personal problem (as long as there is no risk of contamination for others) we are free to take care of ourselves or not.” (patient, man, 58 years old)  “Policing, (no) more freedom for the patient to stop a treatment that seems to be harming him without being observed.” (public, woman, 40 years old)  “A medication such as you're presenting it to me makes me think of forced treatment. If we refuse to take the medication or forget about it will our doctor contact us to explain ourselves?” (patient, woman, 25 years old)  “Policing: no more freedom for the patient to stop a treatment that seems to be harming him without being observed.” (public, woman, 40 years old)  “It's a threat to free will. I know which treatments I can reduce or stop earlier (painkillers, syrup... not antibiotics) and I can perfectly well inform my doctor if I think it is necessary. I prefer that he asks me the question rather than offering to ‘police’ me.” (nurse, woman, 43 years old) | 25 (3.3) | 21 (1.7) | 46 (2.3) | 30 (12.2) |
| Break trust | “The therapeutic relationship is based on a relationship of trust, 'policing' would taint this relationship, it's a question of ethics.” (public, woman, 45 years old)  “I don't need it. I take my medication without making any mistakes. I need the doctor to trust me and I don't want to be treated like a child or 'policed'. This device seems too intrusive. I would not agree to taking it. I would have the impression that my doctor doesn't trust me and wants to keep me under surveillance. I would find it totally inappropriate.” (patient, woman, 28 years old)  “I would think that he doesn't trust me to take a medication, and I might forget about it.” (patient, woman, 79 years old)  “I don't like being kept under surveillance. There is a relationship of trust between my GP, my psychiatrist and me. This would undermine that trust because I would be policed.” (patient, woman, 51 years old)  “This may be perceived by the patient as a lack of trust. You don't pay a visit to the doctor to not take the prescribed treatment. If it is just to find out if I've taken the medication properly, I would be angry because we have to trust each other.” (patient, woman, 57 years old)  “I would certainly agree, but I would like to know why, if he has any doubts about my good compliance in particular.” (patient, woman, 59 years old)  “I'm shocked, we have to believe what patients say, even though we know no one's perfect.” (anaesthetist, woman, 34 years old)  “Ethically it's very questionable, it makes it look like we're ‘spying on’/‘policing’ the patient. What about the patient's freedom? Could such a device really improve patient compliance? A good motivational interview seems more relevant. (It is better to act upstream = prevention and health promotion, therapeutic education, rather than downstream = bad compliance to be ‘reprimanded’) Not to mention that this would put the patient at odds vis-à-vis the doctor in case of poor compliance... And in this case, what about the doctor-patient trust relationship?” (general practitioner, woman, 29 years old)  “Personally, I wouldn't like that kind of policing, I wouldn't willingly offer it to my patients. My patients come to see me because they themselves are looking for a doctor they can talk to in confidence. So I don't have a problem with compliance as long as I listen to their concerns and discuss them in confidence and without judgment with them.” (general practitioner, man, 64 years old) | 36 (4.7) | 39 (3.1) | 63 (3.1) | 147 (59.8) |
| Paternalistic vision of medicine | “This allows the doctor to monitor his patient's seriousness and thus their willingness to heal/be healed.” (public, woman, 29 years old)  “This medication can be useful to avoid omissions that can have consequences (pills, anticoagulants...), but I find that it hinders the confidentiality and freedom of the patient, as if they were being kept under surveillance by the doctor, in a more than paternalistic than egalitarian vision of the relationship...” (patient, woman, 24 years old)  “It's too intrusive, with a ‘big brother is watching you’ effect applied to health...? The doctor has a duty of means +/- of prescription but not to ‘police’ to verify his compliance with his prescription.” (general practitioner, man, 38 years old)  “Paternalistic and intrusive, this type of treatment undermines both the patient's autonomy and the therapeutic alliance. If there are clinical benefits to be expected from it, they will in any case be very meagre in the face of disrespect for the person.” (psychiatrist, man, 26 years old) | 2 (0.3) | 0 | 2 (0.1) | 14 (5.7) |
| Threaten confidentiality | “The confidentiality of this system frightens me and I have no confidence in the system, which will surely be financed by the pharmaceutical laboratories.” (public, woman, 58 years old)  “I would like to have total assurance that the data are inviolable, and are not found in lab listings, for example, or elsewhere!” (public, man, 50 years old)  “I have no trust in the fact that the results are sent to a personal phone where all the apps and Google track what is done on it (leakage of personal results) - I don't trust the security of the Bluetooth system - I'm reluctant if it is to check if the patient is taking their medication every day, like a policeman” (patient, man, 44 years old) | 44 (5.7) | 95 (7.7) | 139 (6.9) | 3 (1.2) |
| Modern medical practice | “If the doctor suggests it to me, I would say to myself that he is looking for efficiency in his treatment.” (public, man, 61 years old)  “I would think that I have a very high-tech doctor.” (patient, woman, 61 years old)  “The doctor's approach can be seen as caring and up-to-date. Offering the latest technologies shows the renewal of his practices” (patient, woman, 19 years old) | 31 (4.1) | 47 (3.8) | 78 (3.9) | 13 (5.3) |
| Empowerment | “Perhaps the person would feel more involved in their care if they take this medication?” (patient, woman, 72 years old)  “There could be an alarm if the medication is not taken. That could also make the person responsible” (public, man, 48 years old)  “We would be more aware of our state of health and the status of the illness because of the smartphone monitoring.” (patient, woman, 47 years old)  “I am willing to take it if it is explained to me, to improve my understanding of the medication being absorbed.” (patient, man, 52 years old)  “I am interested by real-time information on the state of my body.” (public, man, 56 years old)  “I agree to take it because it makes you want to know more about your body” (public, man, 34 years old)  “Depending on the case, we can imagine in certain pathologies a way of involving the patient himself more in his own care. So OK to use this tool, if it is in a context of empowerment.” (general practitioner, man, 31 years old) | 13 (1.7) | 33 (2.7) | 46 (2.3) | 20 (8.1) |
| **Precaution** |  |  |  |  |  |
| Data access restricted to patients only | “I like the concept, but only on the condition that I can also access the data. It would be a good idea to extend it also to personal health monitoring on a daily basis.” (public, woman, 32 years old)  “The data should not be delivered only to the doctor but through an alert system directly to the patient.” (public, woman, 30 years old)  “The principle must be reversed, i.e., the patient must be told that he has forgotten the medication, not the doctor.” (patient, man, 62 years old)  “I take anticoagulants every night and often I don't know if I took them or sometimes I forget. This could be a means of monitoring for me and only for me, not for my doctor.” (patient, man, 68 years old)  “Yes for monitoring compliance if and only if: the data are accessible ONLY to the patient, who can then better manage his treatment, but who decides himself if he wants to show the data to his doctor. If the decision comes from the patient, then this can indeed improve confidence.” (psychiatry, man, 28 years old) | 6 (0.8) | 7 (0.6) | 13 (0.7) | 12 (4.9) |
| Consent | “The patient must be voluntary (except in cases of guardianship, court decision) because otherwise it is an infringement of freedom (patient, woman, 59 years old)  “The idea is a good one, especially if it concerns specific treatments for single, isolated, slightly incapacitated people who may find it comforting to feel monitored and supported if necessary... but this system should not be ‘generalised’ and should only be applied if the ‘sick’ person consents voluntarily.” (public, woman, 70 years old)  “This pushes keeping patient compliance under surveillance into the privacy of the patient, I find that this process is bordering on loss of liberty...but as long as the patient is properly informed of the interest, consents, and has the right to withdraw when he wishes, I don't see a problem.” (patient, woman, 24 years old)  “This device scares me because it could become the norm and no longer be subject to the approval of the patient.” (public, man, 34 years old)  “Will patients take their treatment better knowing that they are being kept under surveillance? But is this a good reason? The manner in which consent is obtained will be important.” (pharmacist, man, 42 years old)  “The issue of use without consent in a programme of care arises: the device seems to me to be difficult to accept for people under a non-consensual measure of care and the patch is probably too intrusive.” (psychiatry, woman, 41 years old) | 4 (0.5) | 10 (0.8) | 14 (0.7) | 26 (10.6) |
| Need a specific legal and ethical framework | “This system must be perfectly supervised.” (patient, woman, 60 years old)  “It's unethical, it's unconstitutional, it's immoral policing.” (public, man, 31 years old)  “I have a problem with the legal ramifications of the concept. For example, in the event of death, insurance companies could use it to avoid compensating the family.” (patient, man, 35 years old)  “It's innovative. But what will the next innovations be? Where will we draw the line under what is acceptable for the monitoring of our health? The first risk that I identify with regard to the reliability of the information is that tomorrow the insurance/CNAM systems will make the reimbursement of expensive therapies conditional on the proper compliance with these therapies, and I see this as the beginning of us being deprived of our freedoms. The debate is on the table.” (pharmacist, man, 31 years old)  “It is very intrusive. This, off the cuff, could conflict with my values and ethics. It could be implemented with a good understanding of the patient, a clearly established interest and the explicit agreement of the patient. And I wonder about efficiency and cost. There again, an ethical question of justice.” (general practitioner, woman, 38 years old) | 2 (0.3) | 2 (0.2) | 4 (0.2) | 3 (1.2) |
| Further investigation of harms/benefits | “I will refuse unless it is experimental for a few weeks and the result is positive in the illness but I think I will think twice about it.” (patient, woman, 61 years old)  “I will discuss with my doctor any studies on the risks involved.” (public, woman, 61 years old)  “This medication must prove itself over time.” (public, man, 69 years old)  “If this device is tested and reliable, I would be interested, but I wouldn't want to be a guinea pig.” (patient, man, 77 years old)  “I am waiting for results on other patients before I take it.” (public, woman, 58 years old)  “Personally, I will not use this device, I would be too afraid of side effects not yet known.” (patient, woman, 63 years old)  “It's still too recent for me to have an opinion on it, we lack the objectivity” (patient, man, 73 years old)  “After 10 years of proven efficacy, I might say yes.” (public, woman, 39 years old)  “I am opposed to the absence of a study on the safety of a drug that emits electromagnetic waves and the certainty that the device cannot be hacked.” (cardiology, man, 32 years old) | 50 (6.5) | 96 (7.8) | 146 (7.3) | 15 (6.1) |
| Restricted use to clinical research | “I would be willing to take it because it can help advance research.”  (public, woman, 53 years old)  “Useful for making sure that the patient is taking his or her medication(s) properly and thus making clinical studies more reliable.” (patient, man, 71 years old)  “If my doctor offers it to me I will refuse, I don't need it, I take my treatment very seriously. But I could accept serving as his ‘guinea pig’ if he needs patients to get their hands on this technology.” (patient, woman, 41 years old)  “I agree because it can help advance research and see that my problem is being properly addressed.” (public, woman, 53 years old)  “I would try voluntarily: if I can make a difference and help my fellow man, I'm a volunteer.” (patient, man, 37 years old)  “Personally, if it can help heal some people, why not try it? A device to be used perhaps more for studies, to help other sick people.” (patient, woman, 36 years old)  “Scientific interest: punctual measurement of the compliance rate according to the ‘categories’ of patients: teenagers, elderly people, people with cognitive disorders... Measuring rate of compliance according to the type of patients would make it possible to better target the least compliant populations, on which efforts should be focused.” (general practitioner, man, 27 years old) | 9 (1.2) | 11 (0.9) | 20 (1) | 12 (4.9) |
| Restricted use for a short time | « I would agree to take such a treatment for a short period to limit the risk of adverse events due to radiation » (public, woman, 23 years old)  « I would agree IF, and only IF, it is a « one shot » treatment, for instance to have a continuous measure of a biological parameter in the body until the sensor is eliminated. (patient, man, 44 years old) | 14 (1.8) | 5 (0.4) | 19 (0.9) | 8 (3.3) |
|  |  |  |  |  |  |
| **Affective attitudes toward digital pills – 8 codes** | | | | | |
| **Positive attitude (total)** |  | 241 (31.4) | 316 (25.5) | 557 (27.8) | 51 (20.7) |
| Enthusiastic | « It's a great step forward in the communication of information, perfectly in line with the times... » (Patient, man, 60 years old)  « This is a major improvement in patient care. The doctor can follow the patient remotely and the patient is no longer at risk of forgetting his or her treatment. » (Patient, man, 43 years old)  « Smart, fast, simple, want to take it for me. » (patient, man, 66 years old) | 100 (13) | 146 (11.8) | 246 (12.2) | 20 (8.1) |
| Curious | « Surprised that such a medication could exist, but modern. » (public, woman, 57 years old)  « Sounds like a good idea. I'm curious to try it. » (patient, man, 54 years old)  « I'm curious to know if the device works. I think it will be useful for monitoring people's health. » (patient, man, 46 years old) | 141 (18.4) | 170 (13.7) | 311 (15.5) | 31 (12.6) |
| **Balanced attitude** | « In my opinion, it should only be used in the case of a very serious illness when you have nothing left to be lost. » (public, woman, 61 years old)  « I'm for moving forward in medicine, but I'm a bit disturbed by this, I think my doctor should explain more to me, only after that I could be reassured, which means I'm not completely against it. » (patient, woman, 76 years old) | 70 (9.1) | 111 (9) | 181 (9) | 41 (16.7) |
| **Negative attitude (total)** |  | 387 (50.5) | 672 (54.3) | 1059 (52.8) | 142 (57.7) |
| Unease | « I think it's a little weird, I don't want to ingest connected pills. » (public, man, 42 years old)  « It makes me uncomfortable: there's an intrusive part... » (patient, woman, 59 years old)  « I think it's a little weird. Wouldn't there be any danger to humans? It seems a little surreal to me! » (public, woman, 65 years old) | 32 (4.2) | 70 (5.7) | 102 (5.1) | 7 (2.9) |
| Skeptical | « This seems to me to be impossible and dangerous. I'm against it. » (public, woman, 73 years old)  « I'm very skeptical of this kind of technology, ingesting a sensor that will circulate in the body seems very surrealistic to me. » (public, woman, 31 years old) | 114 (14.9) | 168 (13.6) | 282 (14.1) | 13 (5.3) |
| Afraid | « I can't have a foreign object inside my body. » (public, man, 51 years old)  « I'm totally against this process. No more freewill. Monitored from all sides. » (patient, woman, 73 years old) | 56 (7.3) | 175 (14.1) | 231 (11.5) | 13 (5.3) |
| Angry | « It's a shame to suggest medications like this, it's an offence to the health of patients ». (patient, man, 46 years old)  « Nonsense, I wouldn't want that for sure. » (patient, woman, 70 years old)  « It's not reassuring to have a digital sensor in a pill: I'm against this kind of stupid idea... » (public, woman, 60 years old) | 178 (23.2) | 255 (20.6) | 434 (21.6) | 108 (43.9) |
| Disgusted | « Strange device, the drugs alone already taste awful so with a digital sensor, I'm curious. » (patient, man, 51 years old)  « Eww!!! None for me! I find it repugnant. » (patient, woman, 57 years old) | 7 (0.9) | 4 (0.3) | 10 (0.5) | 1 (0.4) |
| **Not coded** |  | 69 (9) | 139 (11.2) | 208 (10.4) | 12 (4.9) |
|  |  |  |  |  |  |
| **Perceived opportunity of digital pills - 30 codes** | | | | | |
| **Advantages** |  |  |  |  |  |
| Digital pills are all positive | “Positive: a doctor can have access to all this data, I find it rather reassuring, innovative and I'm for it.” (public, woman, 43 years old)  “It's a simple, concrete and secure device.” (patient, woman, 44 years old)  “Excellent idea. It's convenient and automated. Bluetooth pairing and smartphone are standard equipment nowadays.” (public, man, 38 years old) | 135 (17.6) | 200 (16.2) | 335 (16.7) | 8 (3.3) |
| Precision | “Positive point: the fineness of the analysis” (patient, man, 72 years old)  “It's a good idea because sometimes we mistake our feelings” (public, man, 44 years old)  “It's interesting and practical to have precise data to better understand my pathology and treat with more precision. A direct precision of the physiology of our body for a more precise medication. (public, man, 50 years old)  “The interest of having live information on reactions in the ‘milieu’ of the organisation which, to my knowledge, does not currently exist.” (patient, man, 69 years old)  “Better data and more accurate medicine.” (dentist, man, 25 years old) | 13 (1.7) | 23 (1.9) | 36 (1.8) | 15 (6.1) |
| Remote monitoring | “Frequent and regular monitoring when at present we don't have this on a daily basis - Easy for a patient to use.” (patient, man, 44 years old) | 48 (6.3) | 69 (5.6) | 117 (5.9) | 2 (0.8) |
| Reassuring | “She would feel supported. I believe in technological developments.” (patient, woman, 45 years old)  “It's reassuring to have a daily check-up.” (patient, man, 70 years old)  “In case of forgetfulness or memory loss, it's more than reassuring.” (public, woman, 70 years old)  “It's a very practical advance that can bring comfort to the patient.” (patient, man, 62 years old)  “The person will feel motivated and confident, I'll take it to feel safe.” (public, woman, 54 years old)  “For some patients who are motivated by care, it can even be a certain reassurance, feeling that they are the actors in their illness.” (nurse, woman, 51 years old) | 17 (2.2) | 31 (2.5) | 47 (2.4) | 2 (0.8) |
| Real time information | “Real-time data reliability.” (public, woman, 37 years old)  “The attending physician can monitor his patient's health in real time and make the necessary arrangements.” (patient, woman 60 years old)  “The doctor would see the effect of the medication immediately.” (public, woman, 71 years old)  “This is immediate information allowing modification or intervention.” (patient, man, 73 years old)  “I take my treatment regularly and if there is a problem, the doctor may review the treatment. It's a time saver that can speed up treatment.” (public, woman, 59 years old)  “It is an improvement in surveillance and in the speed of therapeutic adaptation” (cardiologist, man, 32 years old) | 70 (9.1) | 181 (14.6) | 251 (12.5) | 3 (1.2) |
| Facilitate discussion on treatment | “A live connection with the doctor can help to better tailor the treatment without waiting for the next visit or test results. Certainly, a step forward in the patient/doctor relationship.” (public, woman, 6 years old)  “Opportunity to talk to a doctor about his health problems.” 101485238 (public, man, 46 years old)  “A time of therapeutic education will be necessary to understand the use of this type of device which can be the vector of other themes related to the pathology.” (cardiology, woman, 54 years old)  “In the case of anti-Parkinson's: treatments are sometimes very close in frequency and patients are not always objective about their compliance with treatment, which would make it possible to make links in therapeutic education. When we analyse the curves of treatment intake we can discuss with the patient and understand why he takes or does not take the treatment at certain times". (nurse, woman, 34 years old) | 1 (0.1) | 3 (0.2) | 4 (0.2) | 11 (4.5) |
|  |  |  |  |  |  |
| **Disadvantages** |  |  |  |  |  |
| Digital pills are all negative | “No positive points, except to enrich the labs or inform insurance companies.” (patient, man, 66 years old)  “There's too much technology, I'm afraid of the radiation caused by the data transmission. I am suspicious of this kind of practice and sceptical that doctors will be able to monitor the treatment intake of all patients. I would refuse even if my doctor explained the benefits of this device to me.” (patient, woman, 40 years old)  “It's just an aberrant concept. Between the policing, the waves for each medication, the obligation to have a smartphone, the pollution once the medication is eliminated, the extra cost for social security and mutual insurance companies...” (patient, man, 48 years old) | 213 (27.8) | 408 (33) | 621 (31) | 69 (28) |
| We are already surrounded by connected devices | “The idea of having transmitters and receivers directly in my body or on my body does not reassure me. We're already surrounded by too much technology to carry around with us all the time.” (public, woman, 24 years old)  “I do not agree at all: too many connections, not enough responsibility, we become robots. I find that we are making idiots of people with all these connections.” (patient, woman, 61 years old)  “If this continues, we risk being connected on all sides of the body. Example: watch that indicates your sleep time, heart rate etc. + other devices and transmitters, alarm for elderly people etc... It might be ringing all the time, so I'm quite reserved.” 104266081 (patient, man, 66 years old)  “Always connected devices while we hunt down harmful waves for health. I'm already against all electronic systems that ruin our lives.” (patient, woman, 71 years old) | 74 (9.6) | 86 (6.9) | 160 (8) | 17 (6.9) |
| Risk of misuse/hacking of the data | “What are the risks with hacking? Why not a new possibility for crime?” (patient, man, 69 years old)  “I think there's a risk of leakage of medical confidentiality if a hacker hacks into this system or the loss of the smartphone.” (public, man, 47 years old)  “This system can be interesting for the doctor... What about the risks of interception of this sensitive data by banks or insurance companies, for example?” (patient, woman, 53 years old) | 41 (5.3) | 70 (5.7) | 111 (5.5) | 24 (8.5) |
| Too expensive | “It will be an extra cost for social security and mutual insurance companies...” (patient, man, 48 years old)  “It's an inevitable evolution, but I'm not sure it's necessary. The cost must be enormous.” (public, man, 34 years old)  “For a simple medication, I don't see the point, especially since it must be relatively expensive. Poor Social Security! I think it would be too expensive to be prescribed on a large scale. Doctors are obliged to be aware of scientific progress, but they must not want to prescribe it at all costs if it's not necessary, to be fashionable!” (public, woman, 50 years old)  “It seems like an expensive solution to a problem that doesn't warrant this kind of investment.” (patient, man, 73 years old)  Interesting system but I don't think it brings much in the way of benefits for a cost per tablet that will be multiplied. (emergency medicine, man, 27 years old) | 39 (5.1) | 58 (4.7) | 97 (4.8) | 41 (16.7) |
| It will aggravate the feeling of not living “normally” | “I don't see myself being monitored like this, my goal being to live as 'normally' as possible! It's far too constraining and stressful.” (patient, woman, 73 years old) | 3 (0.4) | 3 (0.2) | 6 (0.3) | 1 (0.4) |
| Change the relationship to the own body | “My body wouldn't belong to me anymore. I would have the impression of no longer being the master of our body and that the doctor would not consider me responsible for my life.” (patient, woman, 60 years old)  It is a major change in the relationship to the body and to illness, which I don't see the point of it if it's not to bring each person back even more to be on himself and in self-control, in short, the modern hyper-individual who becomes, thanks to the entrepreneurial machine, an entrepreneur of himself. His own body becomes manageable capital. (general practitioner, woman, 31 years old) | 1 (0.2) | 0 | 1 (0.05) | 1 (0.4) |
| Can’t replace a doctor | “I'm against it, because there's nothing like contact with your doctor, there would be no human contact.” (public, woman, 59 years old)  “A person who has lost his or her abilities can benefit from this device, no better than human assistance.” (patient, woman, 66 years old)  “Being still able to take my medication, I have no interest in undergoing this kind of procedure, which is contrary to my health and ethics. If I am no longer capable, household helpers are there to take care of those who are failing. Let's stop cutting jobs in favour of pharmaceutical and other groups.” (patient, woman, 65 years old)  “This does not replace a doctor.” (patient, man, 35 years old)  “This is the end of medical consultations. There will be no more human contact. I prefer human relationships to machines.” (public, woman, 56 years old) | 19 (2.5) | 30 (2.4) | 49 (2.4) | 18 (7.3) |
| It will break up the patient-physician relationship | “If my doctor offers me that, I'd say he's renounced his Hippocratic oath: instead of treating his patients he's killing them bit by bit, I'm changing doctors.” (patient, man, 46 years old)  “Forbidden to prescribe me anything whatever and I would leave immediately to find another doctor” (public, woman, 52 years old)  “Overall, doctors have to accept that patients are taking their medication, and they save a lot of time by believing that that is the case.” (patient, man, 73 years old)  “Being policed constantly even on health and no ‘proper’ connection to one's doctor.” (public, woman, 46 years old)  “The patient will have less contact with their doctor. And then it means that the doctor no longer trusts the patient.” (public, woman, 59 years old)  “The result is that a patient who forgets his treatment is wrong, a kind of guilt can set in. And what about trust in all this? Our role is to convince the person that the treatment is good for him, if he adheres to our discourse everything will be fine. We also have to listen to his reticence, sometimes totally justified, and adapt to it. For me, this device is absolutely not a solution, and I am not reassured by its use.” (general practitioner, woman, 28 years old)  “It's a bit frightening on the one hand for the patient, who sees personal freedom and responsibility reduced, and on the other hand for the doctor, for whom it's no longer the therapeutic alliance with the patient that is paramount, but rather control.”(general practitioner, woman, 31 years old)  “Questionable benefit, not conducive to the doctor-patient relationship.  Better a healthy relationship and being able to talk openly about compliance, the patient is not obliged to obey the doctor's letter.” (radiology, man, 29 years old) | 19 (2.5) | 38 (3.1) | 57 (2.9) | 26 (10.6) |
|  |  |  |  |  |  |
| **Target population** |  |  |  |  |  |
| Not for me | “At the moment, my memory and my personal organisation are not lacking and I take my medication regularly without forgetting” (patient, man, 74 years old)  No use for me as I currently have no daily treatment to follow and will be able to manage taking the medication.” (public, woman, 31 years old)  “As long as the objective is not specified, it does not seem appropriate to me to leave the day-to-day management of my treatments to a third party.” (patient, man, 72 years old)  “I take my medication regularly. As soon as I forget them I know.” (patient, woman, 45 years old)  “I don't need to be kept under surveillance to take my medication.” (patient, woman, 54 years old)  “That would mean that I was already seriously ill: eek!! Good approach, but may be forgotten by me: reminder by electronic diary.” (public, man, 54 years old)  “Not at the moment, I prefer to take responsibility for complying with my treatment and not to rely on outside control.” (patient, man, 66 years old)  “I think I'm an adult and I can handle myself.” (patient, woman, 69 years old)  “As a doctor I wouldn't tell him I need it because I take my medication. "(psychiatry, man, 30 years old)  “I would refuse. I feel able to follow my treatment to the letter. I would feel policed. “(psychiatry, woman, 27 years old) | 107 (14) | 106 (8.6) | 213 (10.6) | 38 (15.4) |
| Negligent people | “Useful for those who are taking their treatments at random! “(patient, man, 73 years old)  Real-time monitoring by the doctor, especially for the elderly or absent-minded.” (public, woman, 66 years old)  “For a patient who is a little absent-minded, this makes monitoring his treatment possible.” (public, man, 33 years old)  “I have my head in the clouds a little and have to ask my husband whether or not I've been taking my treatment.” (public, woman, 55 years old) | 46 (6) | 39 (3.2) | 84 (4.3) | 0 |
| People who refused to take their treatment | “Treatment surveillance useful for people who against medication.” (public, man, 67 years old)  “It can be good for people who don't want to take their treatments.” (patient, woman, 22 years old)  “Patients in denial with cognitive impairment (general practitioner, man, 38 years old) | 3 (0.4) | 8 (0.6) | 11 (0.5) | 1 (0.4) |
| Elderly people | “For dependent elderly people or for those who do not follow their treatment regularly.” (patient, woman, 70 years old)  “Person who is very old or has lost his mind to think about taking his medication.” (public, man, 55 years old)  “When I am older or I lose my mind to see with the people looking after me and who would send give him a connected chart of the times of taking doses via the internet.” (patient, woman, 83 years old)  “Good idea in geriatrics for important treatments” (orthopaedic surgery, man, 32 years old) | 48 (6.3) | 81 (6.5) | 129 (6.4) | 15 (6.1) |
| Vulnerable people | “This procedure is appropriate for the elderly or disabled or those with serious health problems.” (public, woman, 56 years old)  “Can be very useful for patients who are alone and not very autonomous.” (public, woman, 69 years old)  “Positive if the patient is not able to manage treatment on their own.” (patient, man, 69 years old)  “Perhaps very interesting to monitor the medication of fragile, dependent people...” (patient, woman, 69 years old) | 34 (4.5) | 34 (2.8) | 68 (3.4) | 10 (4.1) |
| People who are under legal constraint | “The person will take his treatment because he is compelled to do so by a court order. He is either a delinquent or a criminal. For someone who has not broken the law, there is no reason to proceed in this way.” (public, man, 39 years old)  “I think that's superfluous in my case. But interesting in the case of a court decision.” (patient, woman, 20 years old)  “I think it can be a good idea for people who are sick (Alzheimer's for example) and forget to take their medication. Or for more complex cases such as people who are dangerous to society and the doctor needs to be sure that the person's treatment has been taken.” (public, woman, 43 years old)  “I would refuse. I feel able to follow my treatment to the letter. I would feel policed. But once again in a psychiatric care programme (constrained but ambulatory) it would be an asset. I can imagine re-hospitalising a patient under duress in a non-observant care programme. This is what already happens when the patient doesn't show up for his injections at the CMP *(medical and psychological consultation centre)*” (psychiatrist, woman, 27 years old) | 1 (0.9) | 6 (0.5) | 7 (0.3) | 2(4.6) |
| People with chronic disease (e.g., diabetes, cardiovascular disease, epilepsy) | “It is a very interesting device for particular monitoring of patients with chronic illnesses.” (public, man, 27 years old)  “Perhaps for people with chronic diseases such as diabetes, etc.” (public, woman, 29 years old)  “"This device would be suitable for certain high-risk pathologies such as heart problems, respiratory in my case, no.” (patient, man, 72 years old)  “The positive points are that for those who have a cardiovascular problem, it is strongly advised to keep them under surveillance.” (public, man, 46 years old)  “Useful for chronic diseases, cancers, depression.” (public, woman, 33 years old) | 10 (1.3) | 31 (2.5) | 41 (2) | 19 (7.8) |
| People with cognitive disorder | “Unless I'm losing my mind, I wouldn't want to be kept under surveillance all the time.” (patient, woman, 77 years old)  “To help people who forget or experience memory loss.”  “It might perhaps be useful for animals, but for humans, I don't see it.” Unless, the person is not autonomous (senile dementia, ...).” (public, woman, 31 years old)  “For the elderly or people with amnesia: allows for better remote monitoring (and less cost for nurses to travel to the home).” (patient, woman, 62 years old)  “I don't see the point in it for me, but for people who are out of their minds, why not? It's not very moral, but why not put it on for people without asking their opinion... (public, woman, 36 years old) | 87 (11.3) | 87 (7) | 174 (8.7) | 29 (11.8) |
| People with mental disorder | “There are some who can't do without control, the fools and the mentally retarded.” (patient, woman, 62 years old)  “For compulsory monitoring of treatment for a mental disorder, where the person may be dangerous without treatment, why not.” (patient, woman, 41 years old)  “I don't see the benefit, if it's for the doc to make sure the patient has taken his medication properly, perhaps for someone suffering from Alzheimer's or schizophrenia who has a duty of care.” (patient, man, 59 years old)  “In my case it's of no benefit! For people with a mental illness or a very serious problem with consequences on their environment it can be of real benefit to monitor!” (public, woman, 66 years old) | 25 (3.3) | 19 (1.5) | 44 (2.2) | 27 (1.3) |
| People with severe diseases | “Process that must be expensive, can be beneficial for serious illnesses that need to be kept under surveillance.” 104873634 (public, woman, 33 years old)  “Of course, it can be very beneficial in the case of serious illness, but I think that it may be an infringement of the patient's freedom, personally: I'm against it.” (public, woman, 52 years old)  “This must be reserved for quite serious pathologies.” (public, man, 18 years old) | 43 (5.6) | 46 (3.7) | 88 (4.4) | 2 (0.8) |
| People with repeated relapse | “For patients who regularly stop their treatment with the recurrence of severe symptoms requiring hospitalisation.” (psychiatry, woman, 58 years old)  “Perhaps of interest to patients who repeatedly decompensate on partial compliance, or pseudo-therapeutic failures.”(psychiatry, woman, 54 years old) | 0 | 0 | 0 | 2 (0.8) |
| People taking complicated treatments | « I’ll take it if necessary, for instance if my treatment would be very complicated. » (public, woman, 67 years old)  « Useful if the dosage regimen change everyday. » (patient, woman, 71 years old) | 11 (1.4) | 11 (0.9) | 22 (1.1) | 2 (0.8) |
| People taking essential treatment | “I only take 2 tablets a day, one in the morning and the other in the evening, so I don't see the point, but I think this treatment can be interesting in the case of heavy treatments where you absolutely must not forget and also at specific times.” (patient, woman, 70 years old)  “For serious pathologies with daily treatment, mandatory.” (public, man, 20 years old) | 31 (4) | 27 (2.2) | 58 (2.9) | 12 (4.9) |
| People taking expensive treatments | The question really arises with some expensive treatments, and some doctors already think that they should stop prescribing them or reimburse poor adherents. (haematology, woman, 31 years old)  “Policing” the patient, not trusting him = limits patient adherence to his treatment? But when there is doubt about compliance with expensive HCV-type treatments, it can certainly be very useful. (pharmacist, woman, 31 years old)  The situations for which this would seem acceptable, however, would be the case of very expensive medication and for a fixed period of time (hepatitis C), situations of injunction to care (and/or for certain severe psychiatric disorders perhaps...) (general practitioner, woman, 41 years old) | 0 | 0 | 0 | 10 (4.1) |
| Beneficial for doctors | “I think it's a good way to save the doctor's time.” (public, man, 57 years old)  “The benefit for the doctor is to monitor the intake of the medication but I don't see it as very useful.” (public, man, 67 years old)  “This allows health professionals to know and monitor their patients' medication intake.” (patient, man, 51 years old) | 34 (4.4) | 45 (3.6) | 79 (3.9) | 17 (6.9) |
| Beneficial for informal caregivers | “For people who are elderly or have Alzheimer's disease. This device is reassuring for relatives and the medical team.” (public, woman, 38 years old)  “In order to attest to the effectiveness of the treatment, which allows the physician to ensure that medication is taken, and to better monitor and evaluate the appropriateness of their diagnosis. And that the data are available to the patient or his family or his counsellor in case of problems and this without any retention.” (public, man, 50 years old) | 2 (0.3) | 5 (0.4) | 7 (0.4) | 0 |
