# Supplementary material for: Acceptability of and Willingness to Take Digital Pills by Patients, the Public, and Health Care Professionals: Qualitative Content Analysis of a Large Online Survey
Source: J Med Internet Res. 2022 Feb 18;24(2):e25597. doi: 10.2196/25597 (PMC8900921; doi:10.2196/25597)
Supplement: Multimedia Appendix 10 [file jmir_v24i2e25597_app10.docx]

# Multimedia Appendix 10: Determinants of the willingness to take digital pills

| **Socio-demographic and health characteristics** | **Total**  **N= 2005** | **Agree to take digital pills**  **N=647** | **Do not agree to take digital pills**  **N=1358** | **Crude OR  [95% CI]** | **Adjusted OR [95% CI]** | **P value for adjusted OR** |
| --- | --- | --- | --- | --- | --- | --- |
| **Sex – n (%)** |  |  |  |  |  |  |
| Woman | 1050 (52.4) | 261 (40.3) | 789 (58.1) | - | - |  |
| Man | 955 (47.6) | 386 (59.7) | 569 (41.9) | 2.06 [1.69; 2.51] * | 1.98 [1.62;2.43]* | 5.9 e-11 |
|  |  |  |  |  |  |  |
| **Age – mean, SD)** | 48.6 (16.4) | 49.0 (16.3) | 48.5 (16.4) | 1.00 [1.00;1.01] | 1.01 [1.00; 1.02] | 0.13 |
|  |  |  |  |  |  |  |
| **Highest level of education – n (%)** |  |  |  |  |  |  |
| Secondary school or under | 95 (4.7)) | 29 (4.5) | 66 (4.9) | 0.92 [0.56;1.49] |  |  |
| Youth training | 386 (19.3) | 127 (19.6) | 259 (19.1) | 0.98 [0.61;1.58] |  |  |
| High school graduate | 471 (23.5) | 148 (22.9) | 323 (23.8) | - |  |  |
| Two-years university degree | 458 (22.8) | 151 (23.3) | 307 (22.6) | 0.92 [0.57;1.48] |  |  |
| Bachelor’s degree (BA, BS) | 341 (17.0) | 106 (16.4) | 235 (17.3) | 1.00 [0.61;1.64] |  |  |
| Master’s degree or beyond | 254 (12.7) | 86 (13.3) | 168 (12.4) | 0.88 [0.53;1.49] |  |  |
|  |  |  |  |  |  |  |
| **Socio-professional category – n (%)** |  |  |  |  |  |  |
| Farmers | 18 (0.9) | 6 (0.9) | 12 (0.9) | 1.04[0.32;3.34] | 1.01[0.25;4.10] | 0.99 |
| Self-employed professional workers | 74(3.7) | 28 (4.3) | 46 (3.5) | 1.21 [0.71;2.05] | 1.25 [0.70;2.21] | 0.45 |
| Senior managers | 199(9.9) | 70 (10.8) | 129 (9.5) | 1.10 [0.78;1.55] | 1.04 [0.68;1.58] | 0.86 |
| Technicians and associate professionals | 305 (15.2) | 108 (16.7) | 197 (14.5) | 1.10 [0.83;1.50] | 1.10 [0.75;1.62] | 0.62 |
| Employees | 349 (17.4) | 93 (14.5) | 254 (18.8) | 0.75 [0.56;0.99] | 0.81 [0.56;1.18] | 0.28 |
| Manual workers | 264 (13.2) | 92 (14.2) | 172 (12.7) | 1.09 [0.78;1.53] | 1.05 [0.70;1.59] | 0.81 |
| Retired people | 559 (27.9) | 184 (28.3) | 375 (27.6) | - | - | - |
| Unemployed | 237 (11.8) | 66 (10.2) | 171 (12.6) | 0.78 [0.54;1.14] | 0.87 [0.56;1.35] | 0.54 |
|  |  |  |  |  |  |  |
| **Population density of residential area – n (%) (inhabitants)** |  |  |  |  |  |  |
| Rural city (< 2000) | 451 (22.5) | 136 (21) | 316 (23.3) | - | - | - |
| [2 000-19 999] | 360 (18.0) | 120 (18.5) | 228 (16.8) | 1.23 [0.90;1.68] | 1.09 [0.79;1.51] | 0.60 |
| [20 000-99 999] | 276 (13.8) | 95 (14.7) | 176 (13.0) | 1.27 [0.90;1.77] | 1.10 [0.77;1.56] | 0.60 |
| ≥ 100 000 | 605 (30.2) | 189 (29.2) | 416 (30.6) | 1.06 [0.80; 1.40] | 0.93 [0.69;1.26] | 0.64 |
| Paris agglomeration | 329 (16.4) | 107 (16.5) | 222 (16.3) | 1.12 [0.81;1.54] | 0.91 [0.65;1.27] | 0.56 |
|  |  |  |  |  |  |  |
| **Has a chronic condition – n (%)** |  |  |  |  |  |  |
| Yes | 767 (38.2) | 271 (41.9) | 496 (36.5) | 1.25 [1.03;1.53] | 1.11 [0.89;1.38] | 0.35 |
| No | 1238 (61.8) | 376 (58.1) | 862 (63.5) | - |  |  |
|  |  |  |  |  |  |  |
| **Duration of the disease – mean (SD)**  **(patients N = 767)** |  | 13.7 (0.7) | 13.5 (0.5) | 1.00 [0.99;1.01] |  |  |
| **Duration of the treatment – mean (SD)**  **(patients N = 767)** |  | 10.2 (0.5) | 10.3 (0.4) | 1.00 [0.99;1.01] |  |  |
|  |  |  |  |  |  |  |
| **Skipped the long-term treatment during the past month – n (%) (patients N = 767)** |  |  |  |  |  |  |
| Never | 495 (64.5) | 170 (62.7) | 325 (65.7) | - |  |  |
| Once a week | 198 (25.8) | 72 (26.6) | 125 (25.2) | 1.01 [0.78;1.55] |  |  |
| Several times a week | 54 (7) | 19 (7) | 35 (7.1) | 1.02 [0.56;1.89] |  |  |
| Almost every day or never started | 20 (2.6) | 10 (3.4) | 10(2) | 1.99 [0.70;5.65] |  |  |
|  |  |  |  |  |  |  |
| **Checking health settings recorded by connected devices – n (%)** |  |  |  |  |  |  |
| Daily | 220 (11.0) | 114 (17.6) | 106 (7.8) | 3.20 [2.33;4.38]* | 3.42 [2.43;4.79]* | 1.6e-12 |
| Weekly | 213 (10.6) | 94 (14.5) | 118 (8.7) | 2.38 [1.72;3.29]* | 2.48 [1.78;3.46]* | 9.7e-08 |
| Monthly | 143 (7.2) | 54 (8.3) | 90 (6.6) | 1.79 [1.23;2.59] | 1.98 [1.34;2.91] | 0.0005 |
| Rarely | 257 (12.8) | 91 (14.1) | 166 (12.2) | 1.64 [1.22;2.21] | 1.76 [1.30;2.40] | 0.0003 |
| Never | 1172(58.4) | 294 (45.4) | 878(64.7) | - |  | - |
|  |  |  |  |  |  |  |
| **Number of visits to a doctor in the past year – n (%)** |  |  |  |  |  |  |
| > 10 times | 208 (10.4) | 70 (10.8) | 138 (10.2) | 1.60 [0.99;2.58] |  |  |
| 5–10 times | 590 (29.4) | 207 (32) | 383 (28.2) | 1.71 (1.14;2.60] |  |  |
| < 5 times | 1046 (52.2) | 332 (21.3) | 714 (52.6) | - |  |  |
| Have not seen a doctor this year | 161 (8) | 38 (5.9) | 123 (9.1) | 1.48 [0.99;2.20] |  |  |
|  |  |  |  |  |  |  |
| **Easiness to talk about the treatment with the doctor (e.g., adherence, adverse events) – n (%)** |  |  |  |  |  |  |
| Easy | 1767 (88.1) | 575 (88.9) | 1192 (87.8) | - | - |  |
| Difficult/do not wish to answer | 228(11.9) | 72 (11.1) | 166 (12.2) | 0.91 [0.65;1.26] | 0.89 [0.63;1.25] | 0.50 |

*Significant after Bonferroni correction (<0.0001)

OR, odds ratio, 95% CI, 95% confidence interval
